# Unveiling the Dynamics of SARS‐CoV‐2 Gamma and Delta Waves in Paraná, Brazil – Delta Displacing a Persistent Gamma Through Alternative Routes of Dispersal

**DOI:** 10.1002/jmv.70318

**Published:** 2025-04-05

**Authors:** Emanuele Gustani‐Buss, Carlos Eduardo Buss, Carlos Alberto Oliveira de Biagi, Isabela Medeiros de Oliveira, Kamila Chagas Peronni, Glauco Akelinghton Freire Vitiello, Bárbara Luisa Fermino, Fernanda Ivanski, Bárbara Mendes Paz Chao, Felipe Francisco Bondan Tuon, Franciele Ani Caovilla Follador, Leia Carolina Lucio, Lirane Elize Defante Ferreto, Marcos Pillegi, Jeane Eliete Laguila Visentainer, Marcia Edilaine Lopes Consolaro, Maria Leandra Terêncio, Dennis Armando Bertolini, Alex Sandro Jorge, Jaime Luis Lopes Rocha, Bruno Zagonel Piovesan, Irina Nastassja Riediger, Diogo Muller Lacerda, Angélica Regina Cappellari, Marco Antonio Largura, Álvaro Largura, Patrik André Barcaro, Vitoria Caroline Tomacheski Schultz Bertol, Marcos Aurélio Pelegrina, Glauco Nonose Negrão, Carla Luiza da Silva, Daniela Frizon Alfieri, Tony Vinicius Moreira Sampaio, Andrea Name Colado Simao, Emerson Carraro, Wilson Araújo Silva, Phillippe Lemey, David Livingstone Alves Figueiredo

**Affiliations:** ^1^ Department of Microbiology and Immunology KU Leuven Leuven Belgium; ^2^ Department of Pediatric Immunology and Infectious Diseases University Medical Centre Utrecht Utrecht Netherlands; ^3^ The Signal Transduction & Metabolism Laboratory (STML) Erasme Campus – Université libre de Bruxelles Brussels Belgium; ^4^ Department of Pediatric Oncology Dana‐Farber Boston Children's Cancer and Blood Disorders Center Boston USA; ^5^ Institute for Cancer Research IPEC Guarapuava Brazil; ^6^ Department of Immunology, Parasitology and General Pathology Center of Biological Sciences (CCB) State University of Londrina (UEL) Londrina Brazil; ^7^ Postgraduate Program in Community Development Midwestern Paraná State University – UNICENTRO Guarapuava Brazil; ^8^ Postgraduate Program in Pharmaceutical Sciences Midwestern Paraná State University – UNICENTRO Guarapuava Brazil; ^9^ Department of Pharmacy Midwestern Paraná State University – UNICENTRO Guarapuava Brazil; ^10^ Laboratory of Emerging Infectious Diseases Pontifícia Universidade Católica do Paraná Curitiba Brazil; ^11^ Postgraduate Program in Applied Health Sciences Western Paraná State University‐UNIOESTE Francisco Beltrão Brazil; ^12^ Departamento de Biologia Estrutural, Molecular e Genética Universidade Estadual de Ponta Grossa, UEPG Ponta Grossa Brazil; ^13^ Universidade Estadual de Maringá, UEM Maringá Brazil; ^14^ Laboratório de Pesquisa em Ciências Médicas (LPCM) Universidade Federal da Integração Latino‐Americana, UNILA Foz do Iguaçu Brazil; ^15^ Departamento de Análises Clínicas e Biomedicina Universidade Estadual de Maringá, UEM Maringá Brazil; ^16^ Laboratório de Diagnóstico Molecular do Hospital Universitário do Oeste do Paraná Universidade Estadual do Oeste do Paraná, UNIOESTE Cascavel Brazil; ^17^ Unimed Laboratório Curitiba Brazil; ^18^ Laboratório Central do Estado do Paraná Curitiba Brazil; ^19^ Biovel Laboratório de Análises e Pesquisas Clínicas Cascavel Brazil; ^20^ Midwestern Paraná State University – UNICENTRO Guarapuava Brazil; ^21^ State University of Ponta Grossa, UEPG Ponta Grossa Brazil; ^22^ State University of Londrina, UEL Londrina Brazil; ^23^ Federal University of Paraná‐UFPR Curitiba Brazil; ^24^ Laboratory of Research in Applied Immunology, Department of Pathology, Clinical Analysis and Toxicology State University of Londrina, UEL Londrina Brazil; ^25^ Virology Laboratory Midwestern Paraná State University – UNICENTRO Guarapuava Brazil; ^26^ Ribeirão Preto Medical School University of São Paulo (USP) Ribeirão Preto Brazil; ^27^ Department of Medicine Midwestern Paraná State University – UNICENTRO Guarapuava Parana Brazil

**Keywords:** Delta‐AY.101, Gamma‐P.1, phylogeography

## Abstract

The Gamma and Delta variants of concern (VOCs) of SARS‐CoV‐2 drove the second and third wave in Brazil and significantly intensified the number of cases and deaths. In this study, we investigate the timeline and origins of the Gamma and Delta variants using a spatiotemporal analysis based on 1508 genomes collected between March and September 2021 from health administrative regions in Paraná state, Brazil. Our findings indicate that community transmission of Gamma‐P.1 began in late 2020, with substantial contributions from the Northeast and North regions. In contrast, our analysis of the Delta‐AY.101 genomes underscored the crucial role of Paraná in national‐level transmission dynamics beginning in late March 2021. At a local level, the movement estimates inferred from the monophyletic clades showed that the Curitiba health region was the primary source for Gamma‐P.1, with a substantial contribution from Londrina. This health‐region also emerged as an important hub for Delta‐AY.101. Our phylogeographical GLM analysis demonstrates that air travel fluxes and population size at the origin of locations were the strongest predictors of shaping SARS‐CoV‐2 dispersal dynamics within Paraná. In addition, viral load analysis suggests that Gamma‐P.1 and Delta‐AY.101 may have maintained a similarly high transmissibility potential throughout the evaluated months, providing insights into the prolonged co‐circulation dynamics. Our study underscores the relevance of understanding SARS‐CoV‐2 introductions and regional circulation contributions at the country level to enhance public health preparedness and strengthen local surveillance programs.

## 1 Introduction

The Severe Acute Respiratory Syndrome Coronavirus 2 (SARS‐CoV‐2) spread globally since December 2019, leading to millions of deaths worldwide [1]. Throughout the pandemic, several SARS‐CoV‐2 lineages have emerged, often carrying mutations that confer adaptive advantages, thereby fueling continuous global circulation [2] and accumulating mutations during the transmission process. This evolutionary process has led to the diversification of variants with varying degrees of transmissibility and immune evasion, significantly influencing the dynamics of the pandemic [3, 4].

The World Health Organization (WHO) has classified variants of concern (VOCs) based on specific mutations, transmissibility rate, pathogenicity, immunogenicity, and their impact on public health interventions. These classification criteria included B1.1.7 (Alpha) that emerged in the United Kingdom, B.1.351 (Beta) in South Africa [5], P.1 (Gamma) in Brazil [6], and B.1.617.2 (Delta) in India [7]. These variants have triggered significant outbreaks that required mitigation measures and intensification of global surveillance [8].

In late 2020, the COVID‐19 epidemic in Brazil was predominantly driven by B.1.128, followed by descendants P.2 and P.1 (VOC Gamma), which emerged in November 2020 [6, 9, 10]. This lineage carried mutations in the spike protein region, conferring increased pathogenicity, transmissibility, and immune evasion capacity. Consequently, the number of cases sharply increased, and P.1 became dominant and widespread in Brazil by early 2021, leading to a significant rise in mortality in the following months [10, 11].

During the intense circulation of P.1 (Gamma) in April 2021, several independent cases of travelers returning to Brazil were diagnosed with the Delta variant in densely populated urban centers associated with international airports. However, Delta only began displacing Gamma in August 2021. In contrast to the global pattern, Delta rapidly displaced Alpha [12]. Studies have shown that the Delta variant exhibits a higher viral load compared to the Alpha lineage, potentially enhancing transmissibility and severity, particularly in unvaccinated individuals [13, 14]. Moreover, Delta sublineages also were associated with a rise in hospital admissions indicating a higher severity in unvaccinated individuals compared to those infected with non‐Delta sublineages and vaccinated individuals [15].

Considering that various factors may shape the heterogeneous viral circulation of respiratory viruses, including mobility, socio‐demographics, population density, herd immunity, and vaccination status, it is crucial to understand their differential impact on case numbers and mortality rates across regions [16, 17]. In this genomic epidemiology study, we aimed to assess the viral diversity circulating at a state‐wide level using a data set of 1508 sequenced positive cases from patients in Paraná, collected from early March 2021 until early September 2021. Through Bayesian spatiotemporal analysis, we investigated the dynamics of two VOCs, Gamma, and Delta, which severely impacted Brazil as witnessed by surges in cases and deaths. Our study delved into the timing of variant introductions, transmission clusters, and routes of dissemination. Specifically, we assessed the repercussions of the healthcare system crisis in Manaus in January on the sustained transmission of the Gamma variant, evaluating the timing, sources, and magnitude of its introductions. For VOC Delta lineages (AY.101 and AY.99.2), we explored potential alternative dispersal routes. Further, to gain insight through transmissibility drivers at the state level, we used a generalized linear model (GLM) extension of discrete phylogeographic diffusion to model discrete location exchange along a phylogeny as a function of various potential predictors, including road travel, air travel, population size, and sample size. In a cohort of 237 sequenced SARS‐CoV‐2 positive cases, comprising Gamma (P.1) and Delta (AY.101) variants, we explored variation in transmissibility over time and between lineages using viral load data. Furthermore, to investigate the late displacement of the Gamma variant, we performed a binomial logistic regression analysis to identify predictors of mortality, providing insights into the factors influencing severity and outcomes associated with different SARS‐CoV‐2 variants.

## 2 Materials and Methods

### 2.1 Data Collection and Ethical Aspects

The study was approved by the ethics committee of the Midwestern Paraná State University registered on the Platform Brazil, CAAE: 45433421.0.0000.0106 (https://plataformabrasil.saude.gov.br).

Nasopharyngeal swab samples were collected from 1508 residents in the state of Paraná and confirmed as positive cases for SARS‐CoV‐2 using real‐time reverse transcriptase polymerase chain reaction (RT‐PCR). Samples were collected from Mestre Clinical Laboratory (Guarapuava, Paraná, Brazil); Regional University Hospital of Maringá Clinical Virology Laboratory of the State University of Maringá, ND Diagnostic Nucleus Laboratory (Maringá, Paraná, Brazil); University Hospital of the State University of Londrina (Londrina Paraná, Brazil); Biovel Laboratory and University Hospital of the State University of Western Paraná (Cascavel, Paraná, Brazil); Department of Structural Biology, Molecular, and Genetics – State University of Ponta Grossa (Ponta Grossa, Paraná, Brazil), Molecular Biology Laboratory of the Federal University of Latin American Integration (Foz do Iguaçu, Paraná, Brazil), Covid‐19 Laboratory of the State University of Western Paraná (Francisco Beltrão, Paraná, Brazil); Unimed laboratory and, Central Public Health Laboratory of Paraná – LACEN/PR (Curitiba, Paraná, Brazil) between March 7, 2021, and September 11, 2021.

Nasopharyngeal swab specimens were collected from all patients using synthetic‐tipped swabs and immediately inserted into a sterile tube containing 1–3 mL of viral transport medium. RNA extraction was carried out using the QIAcube Connect and QIAamp Viral RNA Mini Kit (Qiagen) and eluted in 30 μL, according to the manufacturer's protocol. The positive sample load was selected for next‐generation sequencing analysis.

### 2.2 SARS‐CoV‐2 Amplification and Sequencing

SARS‐CoV‐2 viral genome sequencing was performed using the Illumina CovidSeq SARS‐CoV‐2 Research Panel on the NovaSeq. 6000 platform. Complementary DNA (cDNA) was synthesized from RNA using reverse transcriptase with random hexamers. The synthesized cDNA then underwent two separate PCR reactions to amplify the virus genome present in the sample.

During the library preparation, pooling, and quantification, the amplified products underwent bead‐based tagmentation, where they were fragmented and tagged with adapter sequences. The adapter‐tagged fragments were then subjected to another round of PCR amplification. Subsequently, the indexed tagged libraries were pooled and cleaned using purification beads. The final pooled library product was quantified using the Qubit High Sensitivity dsDNA quantification kit (Invitrogen).

The sequencing‐ready libraries were clustered onto a flow cell and sequenced using sequencing by synthesis chemistry on the Illumina NovaSeq. 6000 sequencing system with NovaSeq SP kit (2 × 100), following the manufacturer's instructions.

### 2.3 Whole‐Genome Processing and Analysis

The raw sequence data underwent quality control analysis using the Illumina pipeline at BaseSpace (https://basespace.illumina.com). Upon evaluating the quality of the obtained reads, trimming was performed using Trimmomatic v.0.39 [18] to remove low‐quality sequences, duplicates, ambiguous bases, and Illumina adapters.

Reads were mapped to the reference SARS‐CoV‐2 genome (GenBank accession number NC_045512.2) using BWA software v0.7.17‐r1188 [19]. Unmapped reads were discarded, and Samtools software v1.11 was used to align reads in random order concerning their position in the reference [20]. Final mutations and indels were called, with low‐quality mutations filtered using the bcftools 1.7‐2 mpileup method [20].

### 2.4 Lineage and Clade Classification

Lineages were assigned from consensus sequences using Pangolin 4.3 (https://pangolin.cog-uk.io) [21]. Genome quality assessment, mutation calling, and clade designation were assigned by Nextclade v3.8.2 [22] (https://clades.nextstrain.org).

### 2.5 Background Sequences

A total of 35 986 genomes for P.1 and 28 790 genomes from AY.99.2 lineage were downloaded from EpiCov on GISAID (https://www.gisaid.org/) on February 14, 2024. For AY.101, all 3616 genomes available on GISAID were included in the further steps (Supporting Information S1).

Genomes from P.1, AY.101, and AY.99.2 were subsampled biweekly based on collection date and Brazilian states, with a maximum number of 100 sequences per state set to maintain diversity, to ensure a balanced representation that avoids overrepresenting specific locations. The filtered sequences were then grouped into regional categories for analysis purposes.

### 2.6 Maximum Likelihood Tree Reconstruction and Temporal Signal Assessment

After subsampling, all sequences were aligned using Nextclade v.3.8 [22]. Phylogenetic trees were reconstructed using IQ‐Tree v.2.3.4 [23], employing a maximum likelihood approach with the GTR+F+R4 as the best‐fit model based on the Bayesian Information Criterion provided by IQ‐Tree's ModelFinder tool. Branch support was assessed using the ultrafast bootstrap approximation (UFBoot) [24]. Before Bayesian time‐measured analyses, the molecular clock signal was examined using Tempest v.1.5.36 [25] to identify and remove outliers based on residual from regression of root‐to‐tip regression.

### 2.7 Time‐Scaled Phylogenetic Tree Reconstruction

To elucidate the diversification of the P.1 and Delta sublineages (AY.101 and AY.99.2) through time, we estimated phylogenetic trees to identify transmission clusters using BEASTv1.10.5 [26]. We employed a strict molecular clock and an HKY85 nucleotide substitution model, along with a nonparametric Bayesian skygrid model as the coalescent tree prior. A total of 200 million Markov Chain Monte Carlo (MCMC) iterations were conducted, combined using LogCombiner [26], and convergence was assessed using Tracer v1.7 (effective sample size [ESS] > 200) [27].

### 2.8 Phylogeographic Discrete Diffusion Analysis for P.1, AY.101, and A.Y.99.2 Variants

A set of 1000 trees were randomly selected from the posterior distribution generated by BEAST for all lineages. Sample locations were designated based on the 27 states and grouped in five Brazilian regions, while for international locations, the five global regions were used as traits in the phylogeographic model. We performed asymmetric phylogeographic reconstruction using Bayesian phylogenetic inference in BEASTv1.10.5 with BEAGLEv4.0.0 library [28]. The pairwise diffusion rates were modeled according to an asymmetric Continuous‐Time Markov Chain process, including a prior on the total number of included rates recommended by Gao et al. [29], while the realizations of this process were estimated as Markov jump counts between states [30]. A sparse set of diffusion rates was estimated using Bayesian stochastic search variable selection (BSSVS) [30]. The MCMC procedure was run for 200 million steps sampling every 10 000 steps. Convergence was verified using TRACER v.1.7 [27], based on ESS values greater than 200 for each parameter, upon which samples were combined using LogCombiner 1.10.4 [26].

The maximum clade credibility tree was summarized in TreeAnnotator [26] and visualized using ggtree [31]. To evaluate sustained transmission potential and sample diversity, we chose only monophyletic clades with posterior state probability (PSP) > 0.9 and with over 20 sequences. Transitions between regions were analyzed using the TreeMarkovJumpHistoryAnalyzer tool to extract the Markov Jumps and time from the posterior tree distribution [32], and only plotted transition between the discrete locations as estimated by the BSSVS analyses supported by Bayes factor (BF) values over *10* using the Circlize R package [33].

### 2.9 Uncovering the Drivers of Viral Transmission in Paraná State

To assess the factors driving the SARS‐CoV‐2 dissemination at the state level, we implemented a GLM extension of discrete phylogeographic diffusion focusing only including Parana genomes for Gamma‐P1 and Delta‐AY.101. Our analysis integrates mobility and demographic data to identify key drivers modeling location exchange along a phylogeny as a function of various potential predictors, including road travel, air travel, population size, and sample size. We used information computed from the road and air data fluxes regarding the period of study, obtained from National Land Transport Agency (ANTT) (https://www.gov.br/antt/) and National Civil Aviation Agency (https://www.gov.br/anac/pt-br/assuntos/dados-e-estatisticas/passageiros), while for population size at origin and destination from IBGE (www.ibge.gov.br).

### 2.10 Assessing the Implications of Viral Load Dynamics

To explore variation in transmissibility, we investigate potential changes in the Cycle threshold (Ct) value over time, which is inversely proportional to viral load [6]. We selected 237 samples positive for SARS‐CoV‐2, diagnosed between March to August 2021, sequenced following the previously described protocol. These samples were obtained using the semi‐quantitative RT‐qPCR method, collected from the same location, and processed according to the same protocol. RNA viral load analysis by RT‐PCR was performed at the Research Laboratory in Applied Immunology, Department of Pathology, Clinical Analysis and Toxicology, State University of Londrina, Londrina, PR, Brazil. The protocol employed the TaqPath COVID‐19 multiplex Real‐Time RT‐PCR test to detect the virus expression using three genes (ORF1ab, N, and S genes), with bacteriophage MS2 serving as a control to confirm the absence of RT‐PCR reaction inhibition. Positive COVID‐19 cases were defined by Ct values ≤ 37 for at least two genes [6]. Continuous variables were assessed for normality distribution via the Shapiro–Wilk test. Subsequently, frequency pairwise comparisons of viral load, quantified by Ct values, were conducted using the Mann–Whitney *U*‐nonparametric test via the Wilcox.rank function.  We assessed the association between categorical variables and Ct values (viral load), during the epidemic growth [34] using log‐Gaussian logistic regression. This analysis tested the effects of sex and age, focusing on changes over the months, including the delay between symptom onset and the collection date, and the presence of symptoms such as anosmia, cough, dyspnea, fever, and myalgia. We first adopted a univariate approach, followed by a multivariate approach employing a generalized model in a stepwise approach based on Akaike criteria selection to obtain the best model. Regression analysis was performed using the glm function in R. In addition, we evaluated mortality predictors through generalized models with a nonbinomial distribution (negative binomial). All the analyses were performed in R version 4.2.1.

## 3 Results

### 3.1 Epidemiological Landscape

**Figure 1:**
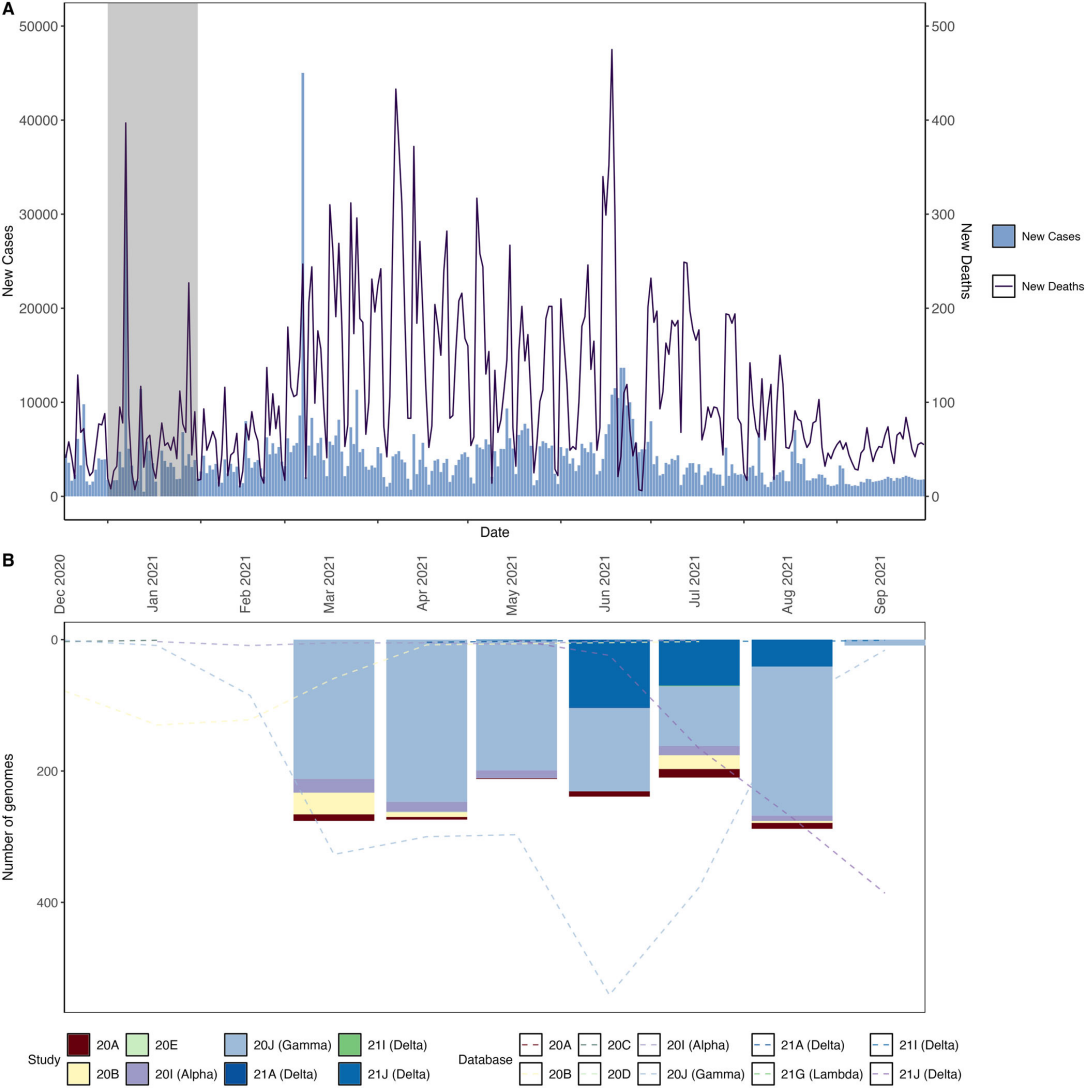
SARS‐CoV‐2 transmission dynamics in Paraná state. (A) Histogram of daily case numbers reported for SARS‐CoV‐2 (light blue) and solid lines (dark blue) summarize the daily numbers of deaths, the gray rectangle represents the month when the vaccination campaign started. (B) Number of genomes sampled during the study from March 05, 2021, to September 09, 2021, bar plots representing the clades identified, and dashed lines illustrate the genomes available on GISAID.

The present study encompasses 1508 positive SARS‐CoV‐2 cases sequenced monthly between March 3, 2021, and September 11, 2021, covering eight municipalities representing the main health administrative regions of Paraná state, Brazil. This study began during the onset of the second wave, marked by an unprecedented number of deaths nationwide and a surge in cases that positioned Brazil as one of the epicenters of the pandemic  in March 2021, as shown in Figure 1. Among the 28 lineages assigned by Pangolin [21], or eight clades classified by Nextclade [22], P.1 Gamma was dominant throughout the sampling period, reaching 69% of the total collection. The Delta variants AY.101 and AY.99.2, which started to increase from June 2021, comprised 12.26% of the total cases (Figure 1).

### 3.2 Phylogeographic Reconstruction of P.1 (Gamma), AY.101 (Delta), and AY.99.2 (Delta)

To investigate the community transmission dynamics of the Gamma variant in Paraná State, we analyzed 934 genomes for P.1 collected over a 7‐month period. In total, 1078 P.1 sequences were sampled from available data on GISAID, spanning all epidemiological weeks across all months, states, and regions. This data set was then used to reconstruct a Bayesian time‐scaled phylogenetic history.

The state reconstruction analysis revealed multiple independent introductions and summarization of viral movements supported the time to the most recent common ancestor was estimated to be on November 29, 2020 (95% CI = November 10, 2020–December 17, 2020) from Northeast, followed by imports from North on January 4, 2021 (95% CI = December 6, 2020–February 24, 2021).

**Figure 2:**
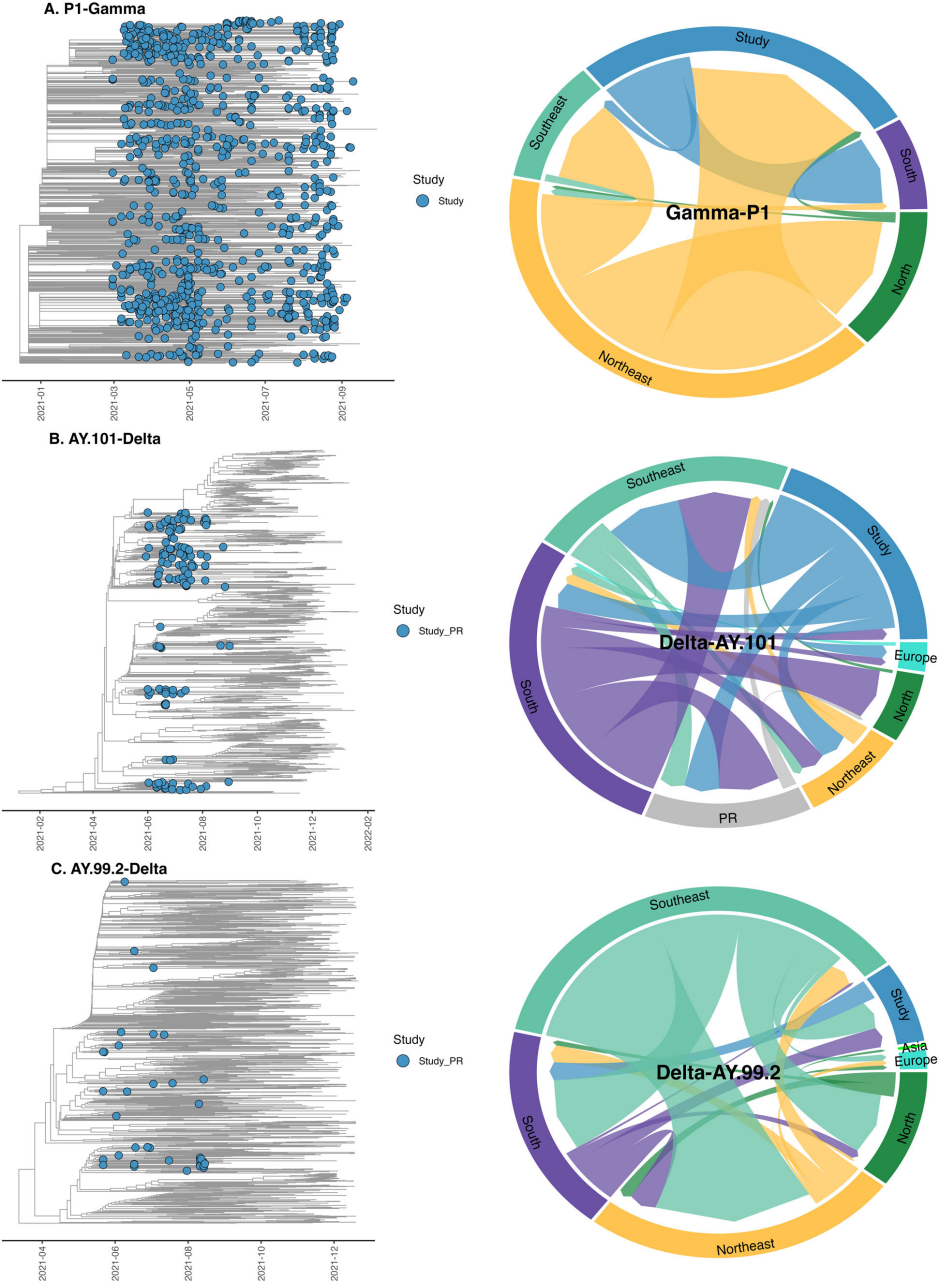
Discrete phylogeographic reconstruction for SARS‐CoV‐2 Gamma and Delta variants circulating in Paraná region. (A) Gamma‐P.1 Maximum clade credibility tree summarized from the Bayesian inference in the left side and right side of the Circular migration flow plots compute the transitions (Markov Jumps with BFs over *10*) between evaluated regions. (B) Delta‐AY.101. (C) Delta‐AY.99.2.

These findings suggest that community transmission started approximately 3 months before the first sampled sequence during a period of escalation in cases and deaths in March 2021. The estimated Markov Jump counts for transmission routes that are supported with BFs over 10, indicate a total of 194 imports, mainly from the Northeast (MJ = 177 events,  95% CI = 171–205) and North (MJ = 7 events, 95% CI = 2–13), and proportionally more exports events to the South (MJ = 74 events, 67–84), and Southeast (MJ = 10 events, 95% CI = 6–17) (Figure 2A, right) (Supporting Information S2: Table S1).

**Figure 3:**
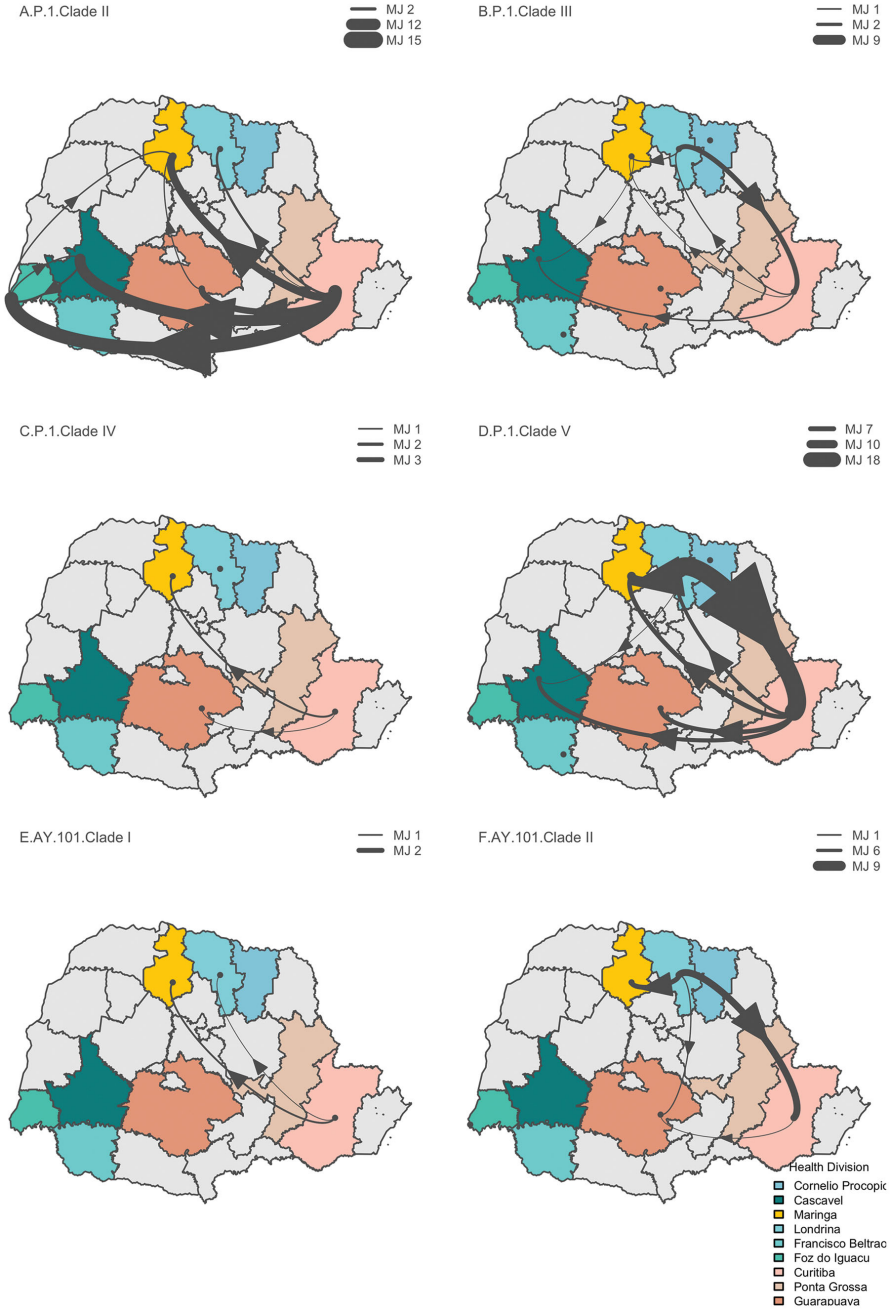
Discrete phylogeographic reconstruction estimates of transmission at the intra‐state level for Gamma‐P.1 and Delta‐AY.101. At health division level of Paraná state, movements reconstruction was performed for clades with more than 18 samples, arrows represent the Markov Jumps transitions (thickness of the lines are proportional to the number) for routes with adjusted Bayes factor support over 3. (A)–(D) Clades with supported transmission routes for Gamma‐P.1. (E) and (F) Clades with supported transmission routes for Delta‐AY.101.

By analyzing the four P.1 monophyletic clades isolated from Paraná, we estimated the dispersal process at the municipality health division level for the majority of nine divisions sampled in this study and distributed across the state. The inference uncovered Curitiba, the capital city region (Figure 3A–D, light salmon), as a main source of dissemination, followed by the Londrina division (Figure 3B–D, light blue) as a secondary hub, both of which seeded cases throughout the state. Clade II (205 samples) encompassed a total of 39 (95% CI = 24–61) transitions mainly from the Curitiba region to Foz do Iguacu (MJ = 19, 95% CI = 11–24), Cascavel (MJ = 16, 95% CI = 8–19), and Maringa (MJ = 12, 95% CI = 5–12), with additional movements from Cascavel to Foz do Iguacu (MJ = 2, 95% CI = 0–6) (Figure 3A). For clade III (33 samples), we identified 13 transitions (95% CI = 0–14), starting from Londrina to Curitiba region (MJ = 10, 95% CI = 0–14), followed by diverse sources of dissemination (Figure 3B). In clade IV (22 samples), we found a pattern mirroring clade II, but reduced in scale in proportion to the sample size, with Curitiba region seeding other regions (Maringa, Londrina, and Guarapuava regions).  In clade V, with the highest representation of within‐state circulation, we estimated a total of five movements from Curitiba to Londrina region (95% CI = 3–10), reaching Maringa (MJ = 9, 95% CI = 4–13), Guarapuava (MJ = 8, 95% CI = 6–10), and Cascavel (MJ = 7, 95% CI = 5–9). Subsequently, we identified bidirectional fluxes from Londrina to Curitiba regions (MJ = 24, 95% CI = 15–35), as well as from Londrina to Maringa region (MJ = 12, 95% CI = 8–16) (Supporting Information S2: Table S2).

To assess the routes of transmission of the Delta variant, one of the main drivers of the third COVID‐19 wave in Brazil, we filtered all available AY.101 genome sequences based on state, region, country, and month, resulting in 778 genomes that were analyzed together with an additional 140 sequences obtained in this study. The phylogenetic tree indicated that Paraná has potentially acted as a major source of transmission for this variant along the South states, with genomes from Paraná clustering into four distinct local clades (PSP > 0.96), suggesting dissemination starting on March 18, 2021 (95% CI = February 17, 2020–April 14, 2021). State reconstruction analyses for this variant indicated international introduction from Europe to the region, establishing a local transmission chain within Paraná State (Figure 2B, left). The transition inference identified exports from Paraná to other regions, including 46 events (95% CI = 41–62), initially directed toward the Southeast (MJ = 19 events, 95% CI = 17–21), South (MJ = 7 events, 95% CI = 5–8), and within Paraná state (MJ = 8 events, 95% CI = 7–11) (Supporting Information S2: Table S3). This observation highlights Paraná's role as a key transmission hub, seeding the primary introductions to other regions of the country  (Figure 2B, right). For the two AY.101 monophyletic clades, the circulation patterns differed from Gamma, with the Londrina region emerging as the main source, underscoring the relevance of this larger urban center as an alternative source. For clade I (18 samples), we observed fewer movements as expected, given the smaller sample size (MJ = 3, 95% CI = 0–9, from Curitiba to Londrina and Maringa regions). Interestingly, in clade II (60 sequences), the Markov jumps estimates were mainly transitions from Londrina to Curitiba (MJ = 11, 95% CI = 8–13) and to Maringa regions (MJ = 7, 95% CI = 5–8) (Supporting Information S2: Table S4). Conversely, the the state reconstruction for the transmission cluster of the AY.99.2 variant, which contributed to the surge in cases and emerged as the dominant lineage in other regions, suggested an origin from the Southeast, on May 19, 2021 (MJ = 95% CI = May 14, 2021–May 25, 2021). The discrete reconstruction indicated the Southeast as a main source of this variant within the country (depicted in green, Figure 2C, right), accounting for 12 events, 95% CI = 10–15 (Supporting Information S2: Table S5).

### 3.3 Drivers of Viral Transmission in Paraná State

**Figure 4:**
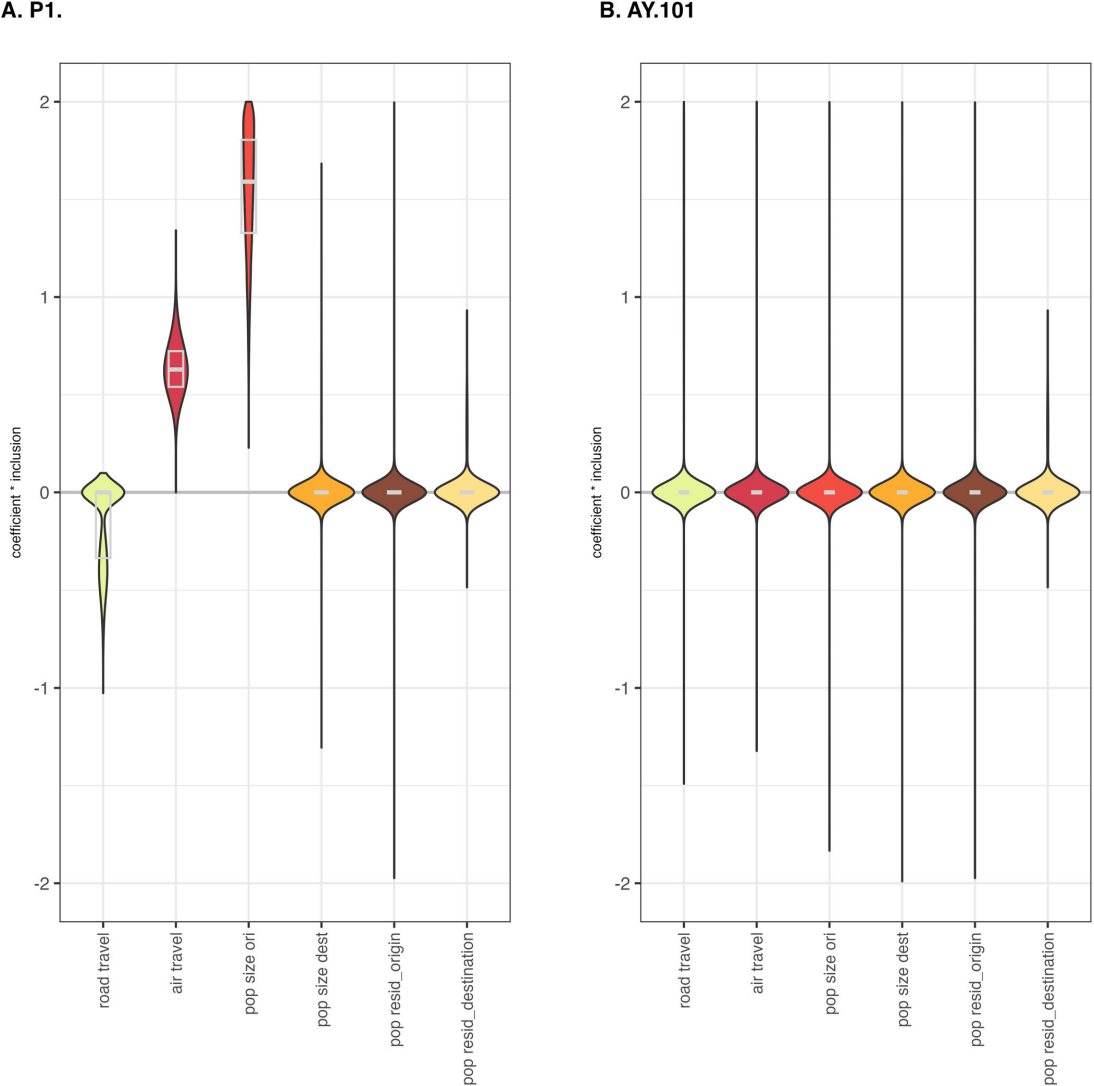
Predictors of SARS‐CoV‐2 lineage movements using a phylogeographic GLM‐diffusion model. The plots summarize the posterior distribution of the product of the coefficient (on a log scale) and the inclusion probability for the predictors (coefficient × inclusion), for the Gamma and Delta lineages (median and quantile estimates), respectively. The predictors tested were road travel, air travel, population size at the origin and destination, and the residuals for regression of population size and genome numbers at the origin and destination. The phylogeographic analysis used cities' health administrative regions as discrete geographic units.

In our Bayesian analysis, we also fit a GLM parameterization of the discrete diffusion process over the clades for both lineages to assess the drivers of viral transmission in Paraná State. We considered road travel, air travel, population size (at both the origin and destination), and residuals for regression of sample sizes against population size as covariates of discrete diffusion rates. Applied to Gamma, this GLM approach demonstrates that air travel fluxes and population size at the origin of locations were the strongest predictors that contributed to shaping SARS‐CoV‐2 dispersal dynamics within Paraná (Figure 4). The results also suggest that sample size did not significantly contribute to the inferred dispersal patterns, indicating that our sampling strategy effectively mitigated potential bias in the reconstructions. For the Delta lineage, we did not identify any significant predictors, likely due to the reduced number of clades represented in the analysis.

### 3.4 Assessing Trends in Viral Load Through Time for P.1 (Gamma) and AY.101 (Delta Sublineage)

**Figure 5:**
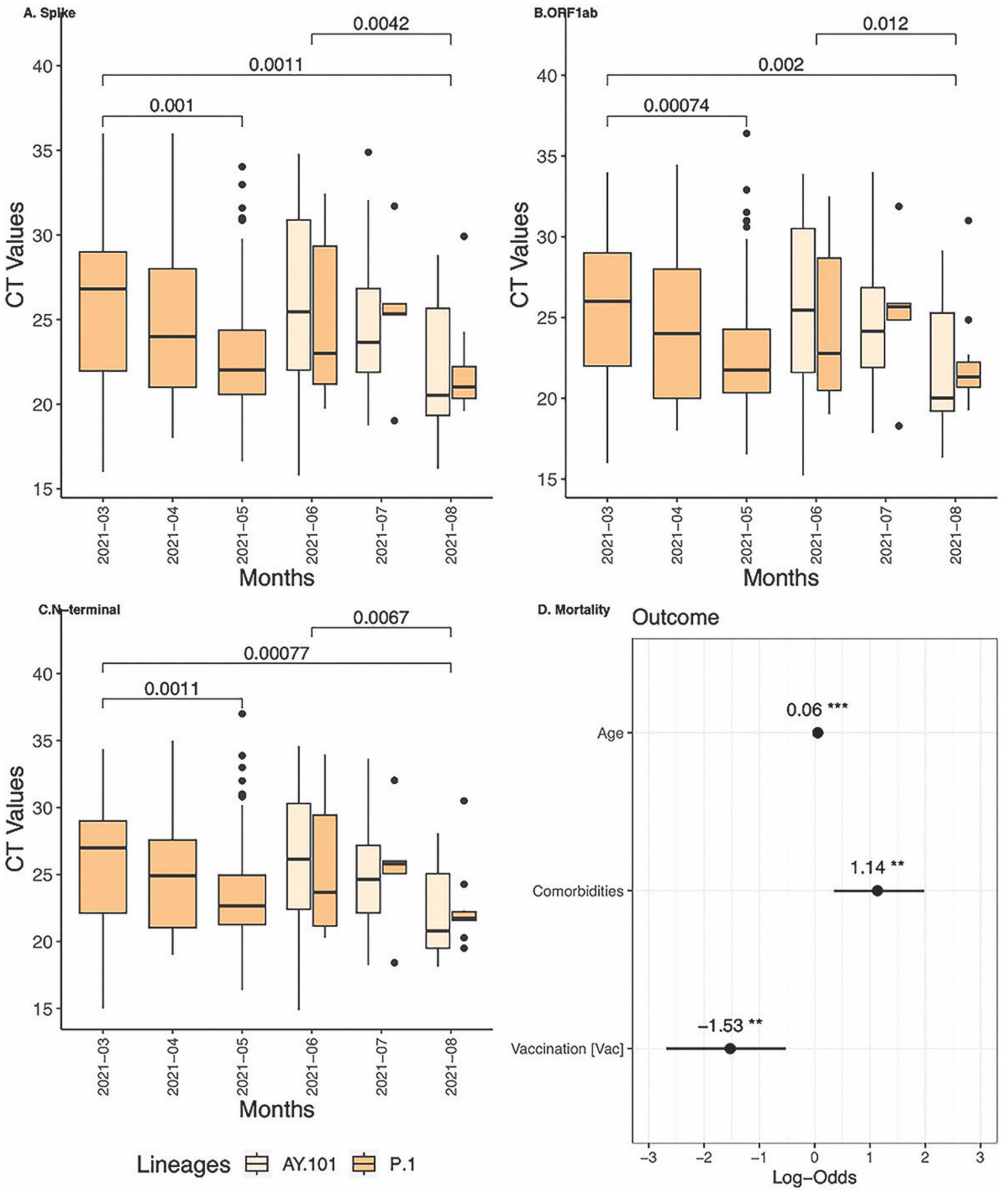
Transmission assessment RT‐PCR Ct values for Spike (S), ORF1ab, and N‐terminal genes for positive SARS‐CoV‐2 cases tested at the hospital in Londrina Paraná from March to August in 2021 and predictors association of clinical outcomes for the cases sequenced. Significant pairwise comparisons performed using the Mann–Whitney test are highlighted with bars. (A) Spike gene. (B) ORF1ab. (C) N‐terminal. (D) Binomial logistic regression for Mortality predictors assessment. The asterisks represent *p*‐values associated with predictors: *p < 0.05; **p < 0.01; ***p < 0.001.﻿﻿﻿﻿

To evaluate potential differences in viral load dynamics between the VOCs through time, we used Ct values obtained from positively diagnosed cases using a SARS‐CoV‐2 quantitative RT‐PCR targeting three genes (Spike, ORF1ab, and N‐terminal regions) from a single laboratory at the University of Londrina (Londrina, Paraná, Brazil) performed from March to August of 2021, minimizing technical variability. The analysis included 237 samples, with 173 samples (genomes) identified as P.1. and 64 samples (genomes) as AY.101. The Mann–Whitney *U*‐test identified a significant decline in viral load (Ct values) for these three genes (Figure 5A–C) for P.1 in the comparative analysis between March and May, and between March and August, and for AY.101 between June and August 2021. In a univariate approach for all genes, regression analysis indicated that the number of days since the onset of symptoms was positively correlated to higher Ct values. Among the five symptoms tested, only three (dyspnea, fever, and myalgia) were associated with lower Ct values.  Similar trends were observed for the months, with May and August showing a significant association with low Ct values. The best‐generalized model identified a significant positive association with the number of days from the first symptom onset, indicating this variable was significantly associated with high Ct values (low viral load). This suggests that there may be differences in viral load during the evaluated period in this study, as the viral load is inversely related to Ct values. These variations could potentially impact transmissibility (Table 1). Both univariate and multivariate approaches did not reveal any significant difference associated with sex, age, and between variants of VOCs (Gamma‐P1 and Delta‐AY.101) (Figure 5).

**Table 1:** Univariate and multivariate linear regression used to test the associations between variables and viral load for three genes: ORF1ab, Spike, and N‐terminal.

	Log of viral load (Orf1ab)		Log of viral load (Spike)		Log of viral load (N)	
	Univariate	Multivariate	Univariate	Multivariate	Univariate	Multivariate
Predictors						
Age	0 (−0.00 to 0.00)	—	0 (−0.00 to 0.00)	—	0 (−0.00 to 0.00)	—
Sex						
Women	3.18 (3.15–3.21)	—	3.18 (3.15–3.21)	—	3.2 (3.17–3.23)	—
Men	−0.01 (−0.06 to 0.03)	—	−0.01 (−0.06 to 0.03)	—	−0.02 (−0.06 to 0.03)	—
Days from symptom onset	0.01 (0.00–0.01)	0.01*** (0.00–0.01)	0.01*** (0.00–0.01)	0.01*** (0.00–0.01)	0.01*** (0.00–0.01)	0.01*** (0.00–0.01)
Symptoms						
Anosmia present	−0.04 (−0.12 to 0.04)	—	−0.04 (−0.12 to 0.04)	−0.04 (−0.12 to 0.04)	−0.03 (−0.11 to 0.05)	—
Cough present	−0.04 (−0.09 to 0.01)	—	−0.04 (−0.08 to 0.01)	−0.04 (−0.08 to 0.01)	−0.03 (−0.08 to 0.01)	—
Dyspnea present	0.07** (0.02–0.11)	—	0.07** (0.02–0.12)	—	0.05* (0.01–0.09)	—
Fever present	−0.06* (−0.11 to 0.01)	−0.05* (−0.10 to 0.00)	−0.05* (−0.10 to 0.00)	—	−0.05* (−0.10 to 0.00)	−0.05* (−0.10 to 0.01)
Myalgia present	−0.09*** (−0.14 to 0.04)	−0.05 (−0.10 to 0.00)	−0.09*** (−0.14 to 0.04)	—	−0.07** (−0.12 to 0.02)	—
Outcome						
Death	−0.02 (−0.07 to 0.03)	—	−0.02 (−0.07 to 0.04)	—	−0.02 (−0.07 to 0.04)	—
Lineages						
Delta	3.19 (3.14–3.23)	—	3.19 (3.14–3.23)	—	3.21 (3.16–3.25)	—
Gamma	−0.02 (−0.08 to 0.03)	—	−0.03 (−0.08 to 0.03)	—	−0.02 (−0.07 to 0.03)	—
Month						
April	−0.05 (−0.14– to 0.03)	−0.07 (−0.15–0.01)	−0.04 (−0.12 to 0.04)	−0.06 (−0.14 to 0.02)	−0.06 (−0.14 to 0.02)	−0.07 (−0.15 to 0.00)
May	−0.11** (−0.17 to 0.04)	−0.11*** (−0.17 to 0.05)	−0.11*** (−0.18 to 0.05)	−0.11*** (−0.17 to 0.05)	−0.10** (−0.16 to 0.04)	−0.10** (−0.16 to 0.04)
June	−0.02 (−0.09 to 0.06)	−0.03 (−0.11 to 0.04)	−0.01 (−0.08 to 0.07)	−0.06 (−0.14 to 0.01)	−0.02 (−0.09 to 0.06)	−0.02 (−0.09 to 0.05)
July	−0.04 (−0.12 to 0.04)	−0.07 (−0.14 to 0.01)	−0.03 (−0.11 to 0.05)	−0.07 (−0.14 to 0.01)	−0.04 (−0.12 to 0.03)	−0.06 (−0.13 to 0.01)
August	−0.15** (−0.24 to 0.05)	−0.16*** (−0.25 to 0.07)	−0.15** (−0.24 to 0.06)	−0.16*** (−0.25 to 0.07)	−0.15*** (−0.24 to 0.06)	−0.16*** (−0.25 to 0.08)

*Note:* “‐” = Not selected or not included in the best model selection based on Akaike's Information Criterion.

Abbreviation: CI, confidence interval.

* *p* < 0.05

** *p* < 0.01

*** *p* < 0.001.

### 3.5 Demographic and Clinical Association

In the analysis focusing on mortality as an outcome, the best‐fit model based on AIC indicated a potential association with the age predictor (OR = 0.06, 95% CI = 0.032–0.08) and the presence of at least one comorbidity, which increased the probability of mortality (OR = 1.14, 95% CI = 0.35–1.98) (Figure 5D). Interestingly, an alternative hypothesis test comparing vaccinated versus unvaccinated individuals in a pooled database encompassing P.1 and AY.101 lineages, suggested a protective effect with decreased mortality among vaccinated individuals, independent of lineage (OR = −1.52, 95% CI = −2.68 to −0.52, *p* = 0.005). This finding supports the efficacy of the immunogenic response against these VOCs (Figure 5D).

## 4 Discussion

The study presented here conducted a comprehensive assessment of the diversity and lineage circulation dynamics of SARS‐CoV‐2 using a cohort of 1508 genomes sampled in Paraná state between March 3, 2021, and September 9, 2021. The analysis identified 28 distinct lineages and eight clades, revealing that 69% of patients were infected with the P.1 (Gamma) variant, while 12.6% were associated with Delta sublineages AY.101 and AY.99.2. By integrating the genomic and spatial data in a phylogeographic approach, we gained insights into the timing of introductions and the sources of those dominant lineages. The analysis underscored an intense Gamma dissemination originating mainly from the Northeast and North regions in late 2020. Notably, genomes from AY.101 sampled in Paraná indicated that this South region played a key role in exporting infections to other regions, pointing at Paraná as a relevant source region for seeding infections across other Brazilian areas located in the Southern region. Our Bayesian analysis unveiled mobility and demographic factors as covariates driving dissemination at the state level. Additionally, the Mann–Whitney test and the Gaussian linear regression showed an increase in viral load (evidenced by a decline in Ct values) over time in both lineages evaluated, suggesting P.1 (Gamma variant) and AY.101 (Delta variant) co‐circulated with a high transmissibility potential [10].

The dominance of the Gamma variant in the study underscored its high transmission potential, leading to an epidemic wave of unprecedented scale, with a sharp increase to 3000 daily deaths in Brazil in late March 2021 [10]. Our phylogeographic inference corroborates early multiple introductions of Gamma from late November 2020 from the Northeast region and from the North in early January 2021 before the collapse of the health system in Amazonas [11, 35]. These findings supported the critical role of the North region in seeding cases to other states following the emergence of the Gamma variant [6]. Moreover, the Markov Jumps transition analysis revealed two main sources, mainly from the Northeast and North, to Paraná, intensifying from late December and peaking in February and March. Subsequently, local transmission was observed, with cases spreading from the South and Southeast regions, indicating widespread transmission across a long‐distance standard, likely fueled by relaxed social distancing measures and nonpharmaceutical interventions [36]. The role of Amazonas as a source has been reported by previous studies, with multiple introductions to most Brazilian regions by mid‐January 2021 [9, 36]. Additionally, our study highlights Paraná as a region exporting infections to other regions to states in the south. Notably, the study highlights the importance of studying routes of dissemination and expanding surveillance to small and mid‐size cities to understand transmission dynamics in a continental country [11].

The phylogeographic inference of Delta sublineages revealed an international introduction followed by local diversification in mid‐March 2021. Genomes of AY.101 from Paraná played a significant role as a source, exporting viral movements to the South and Southeast regions. While we made an attempt to mitigate sampling bias in our discrete trait analyses, we acknowledge that our results need to be interpreted considering factors such as sample size heterogeneities and unsampled data. Reassuringly, a phylogeographic study performed in the south of Brazil based on genomes collected from Rio Grande do Sul is in line with our findings by reporting AY.101 introductions from Paraná [37]. Similar findings suggest that the Delta lineages AY.101 and AY.99.2 dominated COVID‐19 cases across Brazil, except for AY.99.2 in Paraná, as observed in this study, which could suggest a possible competition site, with an efficient transmission chain of AY.101 [37, 38]. The relative transmission ability of SARS‐CoV‐2 has been explored using epidemic models in studies on the co‐transmission of strains. This study suggested that even strains with higher transmissibility may not surpass another strain that has already spread significantly within the population, except in cases where the emerging strain has both stronger transmission and immune escape capabilities [39]. Additionally, transmission of an emerging pathogen may be influenced by factors such as herd immunity, increased vaccination coverage, population density, social interactions, and mobility patterns, as well as the persistent circulation of the P.1 variant, which carries a Spike mutation that enhances transmissibility [40, 41].

Furthermore, the analysis of intra‐state movements for both lineages, based on the monophyletic clades, revealed local transmission sources, suggesting a high degree of interconnectedness. We highlighted intense transmission levels and unveiled a prominent role of transmission between prominent health division regions, the capital city region (Curitiba) and Londrina region, a northern Paraná region for Gamma‐P.1. These imports from a noncapital city region underscore the importance of scaling up surveillance programs to increase the response capacity in small and mid‐sized cities [42, 43].

The two monophyletic Delta‐AY.101 clades, in particular clade II, also support intense transmission dynamics, as well as alternative dispersal routes, including at the intra‐state level, pointing at the Londrina region as a main source. This agrees with previous phylogenetics approaches employed to investigate superspreading events associating outbreaks with healthcare facilities, church services, and terrestrial mobility within the country [44, 45]. Brazil's healthcare system, as  part of its strategy to coordinate responses, decentralizes resources across the states, including the availability of hospital beds, intensive care units, and ventilators. However, these resources are often concentrated in large cities, which, especially during pandemic peaks, face immense pressure as they receive a massive influx of critically ill patients from neighboring towns. This can result in an increased flow of patients from areas with lower population density to those with higher population density [46]. These findings highlight the crucial aspect of passenger transport in fueling transmission during the pandemic and underscore the importance of expanding the local surveillance network [17, 45].

Our phylogeographic reconstructions, employing a GLM framework that incorporates human mobility and population size, yielded significant insights into local transmission dynamics. The results demonstrated a strong correlation between population size at transmission origins and air travel patterns, supporting key region hubs of Gamma‐P1 lineage dissemination within the state. These findings underscore the critical role of population density and mobility networks in driving the spread of the Gamma variant during a pivotal phase of the pandemic. Notably, our results are consistent with reported transmission events that coincided with the temporary relaxation of public health restrictions between mid‐ and late 2020 [11]. These findings emphasize the necessity of evaluating location‐specific characteristics as pivotal determinants of viral dissemination. For instance, one of the health administrative regions, Curitiba, as the capital and most populous city in Paraná, serves as a major transportation and economic hub with extensive domestic and international linkages. Its strong connectivity to other regions of Brazil, such as Southeast and Northeast, that present repeated introductions and sustained transmission may have facilitated the early establishment and onward spread of P.1. The city's high population density and economic activity may have further contributed to accelerated transmission, aligning with previous observations that major metropolitan areas often serve as primary dissemination centers for emerging variants [9].

In particular, considering Delta lineages carried mutations associated with a high transmission potential that could drive a fast replacement. Gamma demonstrates a turnover observed during July and August, also reported for a study in Rio Grande do Sul and Para [37, 47]. This pattern corroborates the events reported in other Brazilian regions and neighboring countries, contrasting with the escalation in Delta cases observed globally [36, 48]. Analysis of trends in SARS‐CoV‐2 Ct values across different months for three genes (S, Orfa1b, and N), indicated a potential increase in transmissibility for P.1 (Gamma lineage) and AY.101 (Delta lineage) across periods of high infection rates. Notably, a significant decline in Ct values was observed comparing March to May and comparing March to August for Gamma, and between June and August for the Delta, suggesting potential fluctuations in transmissibility over time. A similar decline in Ct value (N and E genes) was found in a study exploring P.1 epidemiology in Manaus (Amazonas), where complementary analysis utilizing 942 samples from three time periods (before, during December, and until February 2021) demonstrated significantly lower values, with a persistent decline during periods of heightened circulation [6]. Additionally, a cohort study comprising 244 samples exploring the Gamma viral load dynamics in Rio de Janeiro also observed a slight increase in transmissibility [49]. The lack of a meaningful difference in viral load between Gamma and Delta lineages (AY.101) in the initial months of Delta sublineages circulation, may potentially explain the late replacement (only in September) suggested in previous work. A higher transmissibility of Delta lineages over Gamma lineages was identified from September (during its dominance period), indicating a greater potential for sustained transmission for Delta lineages (for AY.99.2) [50].

In the epidemiologic landscape analysis using binomial logistic regression for the cases sequenced and explored in viral load analysis, the best model did not include lineages as a predictor but indicated that mortality was associated with age and comorbidities while vaccination was associated with a protective effect. Vaccination coverage in Brazil primarily focused on those over 60 years old and those with comorbidities, providing prevention of severe cases. However, a study revealed that in the densely populated South and Midwest regions, the differences between vaccinated and unvaccinated individuals (per 100 000 inhabitants) were higher in the states with higher COVID‐19 incidence rates [51].

The study has some limitations. Given the large number of available genomes for lineages to explore phylogenetic dynamics using a Bayesian approach, we implemented a downsampling strategy to mitigate biases in creating the genome background database. Our GLM‐phylogeographic approach, which tested dissemination predictors, revealed that sample size did not significantly contribute to the inferred dispersal patterns, supporting the success of our strategy at certain levels.

## 5 Conclusions

In summary, our study unveiled the timeline of community transmission in Paraná, identifying the main sources of P.1 Gamma, AY.101, and AY.99.2 variants, underscoring the region as one of the main sources of AY.101 for the whole country. A viral load comparison analysis supports the high transmissibility potential during the co‐circulation of P.1 Gamma and AY.101 variants. These findings contribute to understanding the epidemiological dynamics of sustained SARS‐CoV‐2 transmission variants in Paraná state, Brazil, highlighting the relevance of local genomic surveillance integrated with demographic, epidemiological, and mobility data to unveil the routes of transmission for the future of pandemic interventions.

## Author Contributions

D.L.A.F., E.C., and W.A.S.J. conceived the study and obtained financial support. F.F.B.T., F.A.C.F, L.C.L., L.E.D.F., M.P., J.E.L.V., M.E.LC., M.L.T., D.A.B., A.S.J., J.L.L.R., B.Z.P., I.N.R., D.M.L., A.R.C., M.A.L., A.L., P.A.B., V.C.T.S.B., and A.N.C.S. selected and processed the samples. M.A.P., G.N.N., T.V.M.S., and A.N.C.S. contributed to the collection of public health surveillance data and interpretation. I.M.d.O., K.C.P., G.A.F.V., B.L.F., F.I., and B.M.P.C. sequenced the samples. E.G.B., C.E.B., C.A.O.d.B., and P.L. contributed to the formal data analysis. E.G.B., C.E.B., P.L., and D.L.A.F. drafted the manuscript. All authors contributed to and approved the final version of the manuscript.

## Ethics Statement

The study was approved by ethics committee of  the Midwestern Paraná State University registered on the Platform Brazil, CAAE: 45433421.0.0000.0106 (https://plataformabrasil.saude.gov.br).

## Conflicts of Interest

The authors declare no conflicts of interest.

## Supporting information

Supporting information.

Supporting information.

## Data Availability

All metadata and genomes are available on GISAID's EPICoV database under the EPI_SET_240924xs (visit: 10.55876/gis8.240924xs, also in Supplemental file 1). All BEAST XML files used in this study are publicly available on GitHub: https://github.com/emanuelegustani/unveiling_dynamics_SARS-CoV-2_Gamma_Delta_variants.

## References

[1] F. Wu , S. Zhao , B. Yu , et al., “Author Correction: A New Coronavirus Associated With Human Respiratory Disease in China,” Nature 580, no. 7803 (2020): E7, 10.1038/s41586-020-2202-3. 32296181 PMC7608129

[2] WHO . WHO COVID‐19 Dashboard (WHO, 2024), https://data.who.int/dashboards/covid19/cases.

[3] C. M. Morang'a , J. M. Ngoi , J. Gyamfi , et al., “Genetic Diversity of SARS‐CoV‐2 Infections in Ghana From 2020–2021,” Nature Communications 13, no. 1 (2022): 2494, 10.1038/s41467-022-30219-5. PMC9076825 35523782

[4] P. V. Markov , M. Ghafari , M. Beer , et al., “The Evolution of SARS‐CoV‐2,” Nature Reviews Microbiology 21, no. 6 (2023): 361–379, 10.1038/s41579-023-00878-2. 37020110

[5] H. Tegally , E. Wilkinson , M. Giovanetti , et al., “Detection of a SARS‐CoV‐2 Variant of Concern in South Africa,” Nature 592, no. 7854 (2021): 438–443, 10.1038/s41586-021-03402-9. 33690265

[6] N. R. Faria , T. A. Mellan , C. Whittaker , et al., “Genomics and Epidemiology of the P.1 SARS‐CoV‐2 Lineage in Manaus, Brazil,” Science 372, no. 6544 (2021): 815–821, 10.1126/science.abh2644. 33853970 PMC8139423

[7] P. Mlcochova , S. A. Kemp , M. S. Dhar , et al., “SARS‐CoV‐2 B.1.617.2 Delta Variant Replication and Immune Evasion,” Nature 599, no. 7883 (2021): 114–119, 10.1038/s41586-021-03944-y. 34488225 PMC8566220

[8] N. Zhao , N. Zhou , H. Fan , et al., “Mutations and Phylogenetic Analyses of SARS‐CoV‐2 Among Imported COVID‐19 From Abroad in Nanjing, China,” Frontiers in Microbiology 13 (2022): 1, 10.3389/fmicb.2022.851323. PMC8969601 35369437

[9] D. S. Candido , I. M. Claro , J. G. de Jesus , et al., “Evolution and Epidemic Spread of SARS‐CoV‐2 in Brazil,” Science 369, no. 6508 (2020): 1255–1260, 10.1126/science.abd2161. 32703910 PMC7402630

[10] F. G. Naveca , V. Nascimento , V. Souza , et al., “Spread of Gamma (P.1) Sub‐Lineages Carrying Spike Mutations Close to the Furin Cleavage Site and Deletions in the N‐Terminal Domain Drives Ongoing Transmission of SARS‐CoV‐2 in Amazonas, Brazil,” Microbiology Spectrum 10, no. 1 (2022): e0236621, 10.1128/spectrum.02366-21. 35196783 PMC8865440

[11] T. Gräf , G. Bello , F. G. Naveca , et al., “Phylogenetic‐Based Inference Reveals Distinct Transmission Dynamics of SARS‐CoV‐2 Lineages Gamma and P.2 in Brazil,” iScience 25, no. 4 (2022): 104156, 10.1016/j.isci.2022.104156. 35368908 PMC8957357

[12] A. Bolze , S. Luo , S. White , et al., “SARS‐CoV‐2 Variant Delta Rapidly Displaced Variant Alpha in the United States and Led to Higher Viral Loads,” Cell Reports Medicine 3, no. 3 (2022): 100564, 10.1016/j.xcrm.2022.100564. 35474739 PMC8922438

[13] R. Costa , B. Olea , M. A. Bracho , et al., “RNA Viral Loads of SARS‐CoV‐2 Alpha and Delta Variants in Nasopharyngeal Specimens at Diagnosis Stratified by Age, Clinical Presentation and Vaccination Status,” Journal of Infection 84, no. 4 (2022): 579–613, 10.1016/j.jinf.2021.12.018. PMC8694784 34953901

[14] C. Huai Luo , C. Paul Morris , J. Sachithanandham , et al., “Infection With the Severe Acute Respiratory Syndrome Coronavirus 2 (SARS‐CoV‐2) Delta Variant Is Associated With Higher Recovery of Infectious Virus Compared to the Alpha Variant in Both Unvaccinated and Vaccinated Individuals,” Clinical Infectious Diseases 75, no. 1 (2022): e715–e725, 10.1093/cid/ciab986. 34922338 PMC8903351

[15] T. Nyberg , K. Harman , A. Zaidi , et al., “Hospitalization and Mortality Risk for COVID‐19 Cases With SARS‐CoV‐2 AY.4.2 (VUI‐21OCT‐01) Compared to Non‐AY.4.2 Delta Variant Sublineages,” Journal of Infectious Diseases 226, no. 5 (2022): 808–811, 10.1093/infdis/jiac063. 35184201 PMC8903446

[16] N. D. Grubaugh , J. T. Ladner , P. Lemey , et al., “Tracking Virus Outbreaks in the Twenty‐First Century,” Nature Microbiology 4, no. 1 (2018): 10–19, 10.1038/s41564-018-0296-2. PMC6345516 30546099

[17] P. Lemey , S. L. Hong , V. Hill , et al., “Accommodating Individual Travel History and Unsampled Diversity in Bayesian Phylogeographic Inference of SARS‐CoV‐2,” Nature Communications 11, no. 1 (2020): 5110, 10.1038/s41467-020-18877-9. PMC7547076 33037213

[18] A. M. Bolger , M. Lohse , and B. Usadel , “Trimmomatic: A Flexible Trimmer for Illumina Sequence Data,” Bioinformatics 30, no. 15 (2014): 2114–2120, 10.1093/bioinformatics/btu170. 24695404 PMC4103590

[19] H. Li and R. Durbin , “Fast and Accurate Short Read Alignment With Burrows‐Wheeler Transform,” Bioinformatics 25, no. 14 (2009): 1754–1760, 10.1093/bioinformatics/btp324. 19451168 PMC2705234

[20] P. Danecek , J. K. Bonfield , J. Liddle , et al., “Twelve Years of SAMtools and BCFtools,” GigaScience 10, no. 2 (2021): 1–4, 10.1093/gigascience/giab008. PMC7931819 33590861

[21] Á. O'Toole , V. Hill , O. G. Pybus , et al., “Tracking the International Spread of SARS‐CoV‐2 Lineages B.1.1.7 and B.1.351/501Y‐V2,” Wellcome Open Research 6 (2021): 121, 10.12688/wellcomeopenres.16661.1. 34095513 PMC8176267

[22] I. Aksamentov , C. Roemer , E. Hodcroft , and R. Neher , “Nextclade: Clade Assignment, Mutation Calling and Quality Control for Viral Genomes,” Journal of Open Source Software 6, no. 67 (2021): 3773, 10.21105/joss.03773.

[23] L. T. Nguyen , H. A. Schmidt , A. von Haeseler , and B. Q. Minh , “IQ‐TREE: A Fast and Effective Stochastic Algorithm for Estimating Maximum‐Likelihood Phylogenies,” Molecular Biology and Evolution 32, no. 1 (2015): 268–274, 10.1093/molbev/msu300. 25371430 PMC4271533

[24] D. T. Hoang , O. Chernomor , A. von Haeseler , B. Q. Minh , and L. S. Vinh , “UFBoot2: Improving the Ultrafast Bootstrap Approximation,” Molecular Biology and Evolution 35, no. 2 (2018): 518–522, 10.1093/molbev/msx281. 29077904 PMC5850222

[25] A. Rambaut , T. T. Lam , L. Max Carvalho , and O. G. Pybus , “Exploring the Temporal Structure of Heterochronous Sequences Using TempEst (Formerly Path‐O‐Gen),” Virus Evolution 2, no. 1 (2016): vew007, 10.1093/ve/vew007. 27774300 PMC4989882

[26] M. A. Suchard , P. Lemey , G. Baele , D. L. Ayres , A. J. Drummond , and A. Rambaut , “Bayesian Phylogenetic and Phylodynamic Data Integration Using BEAST 1.10,” Virus Evolution 4, no. 1 (2018): vey016, 10.1093/ve/vey016. 29942656 PMC6007674

[27] A. Rambaut , A. J. Drummond , D. Xie , G. Baele , and M. A. Suchard , “Posterior Summarization in Bayesian Phylogenetics Using Tracer 1.7,” Systematic Biology 67, no. 5 (2018): 901–904, 10.1093/sysbio/syy032. 29718447 PMC6101584

[28] D. L. Ayres , M. P. Cummings , G. Baele , et al., “BEAGLE 3: Improved Performance, Scaling, and Usability for a High‐Performance Computing Library for Statistical Phylogenetics,” Systematic Biology 68, no. 6 (2019): 1052–1061, 10.1093/sysbio/syz020. 31034053 PMC6802572

[29] J. Gao , M. R. May , B. Rannala , and B. R. Moore , “Model Misspecification Misleads Inference of the Spatial Dynamics of Disease Outbreaks,” Proceedings of the National Academy of Sciences 120, no. 11 (2023): e22139131200, 10.1073/pnas.2213913120. PMC10089176 36897983

[30] P. Lemey , A. Rambaut , A. J. Drummond , and M. A. Suchard , “Bayesian Phylogeography Finds Its Roots,” PLoS Computational Biology 5, no. 9 (2009): e1000520, 10.1371/journal.pcbi.1000520. 19779555 PMC2740835

[31] G. Yu , “Using ggtree to Visualize Data on Tree‐Like Structures,” Current Protocols in Bioinformatics 69, no. 1 (2020): e96, 10.1002/cpbi.96. 32162851

[32] P. Lemey , N. Ruktanonchai , S. L. Hong , et al., “Untangling Introductions and Persistence in COVID‐19 Resurgence in Europe,” Nature 595, no. 7869 (2021): 713–717, 10.1038/s41586-021-03754-2. 34192736 PMC8324533

[33] Z. Gu , L. Gu , R. Eils , M. Schlesner , and B. Brors , “ *circlize* Implements and Enhances Circular Visualization in R,” Bioinformatics 30, no. 19 (2014): 2811–2812, 10.1093/bioinformatics/btu393. 24930139

[34] M. Marks , P. Millat‐Martinez , D. Ouchi , et al., “Transmission of COVID‐19 in 282 Clusters in Catalonia, Spain: A Cohort Study,” Lancet Infectious Diseases 21, no. 5 (2021): 629–636, 10.1016/S1473-3099(20)30985-3. 33545090 PMC7906723

[35] M. Giovanetti , S. N. Slavov , V. Fonseca , et al., “Genomic Epidemiology of the SARS‐CoV‐2 Epidemic in Brazil,” Nature Microbiology 7, no. 9 (2022): 1490–1500, 10.1038/s41564-022-01191-z. PMC9417986 35982313

[36] I. Arantes , G. Bello , V. Nascimento , et al., “Comparative Epidemic Expansion of SARS‐CoV‐2 Variants Delta and Omicron in the Brazilian State of Amazonas,” Nature Communications 14, no. 1 (2023): 2048, 10.1038/s41467-023-37541-6. PMC10089528 37041143

[37] T. R. y Castro , B. C. Piccoli , A. A. Vieira , et al., “Introduction, Dispersal, and Predominance of SARS‐CoV‐2 Delta Variant in Rio Grande do Sul, Brazil: A Retrospective Analysis,” Microorganisms 11, no. 12 (2023): 2938, 10.3390/microorganisms11122938. 38138081 PMC10745878

[38] P. L. C. Fonseca , F. R. R. Moreira , R. M. de Souza , et al., “Tracking the Turnover of SARS‐CoV‐2 VOCs Gamma to Delta in a Brazilian State (Minas Gerais) With a High‐Vaccination Status,” Virus Evolution 8, no. 2 (2022): veac064, 10.1093/ve/veac064. 35996592 PMC9384558

[39] J. Chen , C. Gu , Z. Ruan , and M. Tang , “Competition of SARS‐CoV‐2 Variants on the Pandemic Transmission Dynamics,” Chaos, Solitons & Fractals 169 (2023): 113193, 10.1016/j.chaos.2023.113193. 36817403 PMC9915129

[40] F. G. Naveca , V. Nascimento , V. C. de Souza , et al., “COVID‐19 in Amazonas, Brazil, Was Driven by the Persistence of Endemic Lineages and P.1 Emergence,” Nature Medicine 27, no. 7 (2021): 1230–1238, 10.1038/s41591-021-01378-7. 34035535

[41] V. B. Franceschi , G. D. Caldana , A. de Menezes Mayer , et al., “Genomic Epidemiology of SARS‐CoV‐2 in Esteio, Rio Grande do Sul, Brazil,” BMC Genomics 22, no. 1 (2021): 371, 10.1186/s12864-021-07708-w. 34016042 PMC8136996

[42] H. Tegally , J. E. San , M. Cotten , et al., “The Evolving SARS‐CoV‐2 Epidemic in Africa: Insights From Rapidly Expanding Genomic Surveillance,” Science 378, no. 6615 (2022): eabq5358, 10.1126/science.abq5358. 36108049 PMC9529057

[43] J. F. Oliveira , A. L. Alencar , M. C. L. S. Cunha , et al., “Human Mobility Patterns in Brazil to Inform Sampling Sites for Early Pathogen Detection and Routes of Spread: A Network Modelling and Validation Study,” Lancet Digital Health 6, no. 8 (2024): e570–e579, 10.1016/S2589-7500(24)00099-2. 39059889

[44] H. A. C. M. Voeten , R. S. Sikkema , M. Damen , et al., “Unraveling the Modes of Transmission of Severe Acute Respiratory Syndrome Coronavirus 2 (SARS‐CoV‐2) During a Nursing Home Outbreak: Looking Beyond the Church Superspreading Event,” Clinical Infectious Diseases 73, no. Suppl_2 (2021): S163–S169, 10.1093/cid/ciaa1664. 33119065 PMC7665385

[45] B. Faucher , C. E. Sabbatini , P. Czuppon , et al., “Drivers and Impact of the Early Silent Invasion of SARS‐CoV‐2 Alpha,” Nature Communications 15, no. 1 (2024): 2152, 10.1038/s41467-024-46345-1. PMC10925057 38461311

[46] A. Bigoni , A. M. Malik , R. Tasca , et al., “Brazil's Health System Functionality Amidst of the COVID‐19 Pandemic: An Analysis of Resilience,” Lancet Regional Health ‐ Americas 10, no. 1 (2022), 10.1016/j.lana.2022.100222. PMC8896985 35284904

[47] C. T. Pinho , A. F. Vidal , T. C. Negri Rocha , et al., “Transmission Dynamics of SARS‐CoV‐2 Variants in the Brazilian State of Pará,” Frontiers in Public Health 11 (2023): 1186463, 10.3389/fpubh.2023.1186463. 37790714 PMC10543262

[48] J. S. Gularte , M. S. da Silva , A. C. S. Mosena , et al., “Early Introduction, Dispersal and Evolution of Delta SARS‐CoV‐2 in Southern Brazil, Late Predominance of AY.99.2 and AY.101 Related Lineages,” Virus Research 311 (2022): 198702, 10.1016/j.virusres.2022.198702. 35104582 PMC8800493

[49] F. R. R. Moreira , M. D'arc , D. Mariani , et al., “Epidemiological Dynamics of SARS‐CoV‐2 VOC Gamma in Rio de Janeiro, Brazil,” Virus Evolution 7, no. 2 (2021): veab087, 10.1093/ve/veab087. 34725568 PMC8522364

[50] J. P. Silva , A. B. de Lima , L. B. Alvim , et al., “Delta Variant of SARS‐CoV‐2 Replacement in Brazil: A National Epidemiologic Surveillance Program,” Viruses 14, no. 5 (2022): 847, 10.3390/v14050847. 35632589 PMC9143796

[51] C. V. B. Santos , T. G. Noronha , G. L. Werneck , C. J. Struchiner , and D. A. M. Villela , “Estimated COVID‐19 Severe Cases and Deaths Averted in the First Year of the Vaccination Campaign in Brazil: A Retrospective Observational Study,” Lancet Regional Health – Americas 17 (2023): 100418, 10.1016/j.lana.2022.100418. 36575682 PMC9779315

