# Supplementary material for: Unveiling the Dynamics of SARS‐CoV‐2 Gamma and Delta Waves in Paraná, Brazil – Delta Displacing a Persistent Gamma Through Alternative Routes of Dispersal
Source: J Med Virol. 2025 Apr 5;97(4):e70318. doi: 10.1002/jmv.70318 (PMC11971956; doi:10.1002/jmv.70318)
Supplement: Supplementary file 1 — Supporting information. [file JMV-97-e70318-s002.pdf]

SUPPLEMENTAL TABLE

Data Availability

GISAID Identifier:	EPI_SET_240924mz
doi:	10.55876/gis8.240924mz

All genome sequences and associated metadata in this dataset are published in GISAID's EpiCoV database. To view the contributors of each individual sequence with details such as accession number, Virus name, Collection date, Originating Lab and Submitting Lab and the list of Authors, visit 10.55876/gis8.240924mz

Data Snapshot

- EPI_SET_240924mz is composed of 2,894 individual genome sequences.
- The collection dates range from 2020-12-17 to 2022-01-21;
- Data were collected in 26 countries and territories;
- All sequences in this dataset are compared relative to hCoV-19/Wuhan/WIV04/2019 (WIV04), the official reference sequence employed by GISAID (EPI_ISL_402124). Learn more at https://gisaid.org/WIV04.
