# Supplementary material for: Unveiling the Dynamics of SARS‐CoV‐2 Gamma and Delta Waves in Paraná, Brazil – Delta Displacing a Persistent Gamma Through Alternative Routes of Dispersal
Source: J Med Virol. 2025 Apr 5;97(4):e70318. doi: 10.1002/jmv.70318 (PMC11971956; doi:10.1002/jmv.70318)
Supplement: Supplementary file 2 — Supporting information. [file JMV-97-e70318-s001.docx]

**Supplementary material**

**Unveiling the Dynamics of SARS-CoV-2 Gamma and Delta Waves in Paraná, Brazil - Delta displacing a persistent Gamma through alternative routes of dispersal**

Emanuele Gustani-Buss^1,^†; Carlos Eduardo Buss ^2^; Carlos Alberto Oliveira de Biagi^3^; Isabela Medeiros de Oliveira^4^; Kamila Chagas Peronni^4^; Glauco Akelinghton Freire Vitiello^5^, Bárbara Luisa Fermino^6^; Fernanda Ivanski^7,^; Bárbara Mendes Paz Chao^8^; Felipe Francisco Bondan Tuon^9^; Franciele Ani Caovilla Follador^10^; Leia Carolina Lucio^10^; Lirane Elize Defante Ferreto^10^; Marcos Pillegi^11^; Jeane Eliete Laguila Visentainer^12^; Marcia Edilaine Lopes Consolaro^12^; Maria Leandra Terêncio^13^; Dennis Armando Bertolini^14^; Alex Sandro Jorge^15^; Jaime Luis Lopes Rocha^16^; Bruno Zagonel Piovesan^16^; Irina Nastassja Riediger^17^; Diogo Muller Lacerda^18^; Angélica Regina Cappellari^18^; Marco Antonio Largura^18^; Álvaro Largura (*In Memoriam*)^18^; Patrik André Barcaro^18^; Vitoria Caroline Tomacheski Schultz Bertol^18^; Marcos Aurélio Pelegrina^19^; Glauco Nonose Negrão^19^; Carla Luiza da Silva^20^, Daniela Frizon Alfieri^21^; Tony Vinicius Moreira Sampaio^22^; Andrea Name Colado Simao^23^; Emerson Carraro^24^; Wilson Araújo Silva Jr.^25^; Phillippe Lemey^1^*†; David Livingstone Alves Figueiredo^26^*†

†Correspondent author.

***** These authors contributed equally to this work and are co-senior authors.

Table of contents

**Table S1**. Markov jumps inferred by discrete phylogeographic inference for Gamma-P1……………2

**Table S2.** Summary of Markov jumps inferred for the Clusters of transmission for Gamma-P.1 in Parana in a discrete phylogeographic analysis at level of health administrative regions………………3

**Table S3.** Markov jumps inferred by discrete phylogeographic inference for Delta-AY.101….…...…9

**Table S4.** Summary of Markov jumps inferred for the Clusters of transmission for Delta-AY.101 in Parana in a discrete phylogeographic analysis at level of health administrative regions………..……12

**Table S5.** Markov jumps inferred by discrete phylogeographic inference for Delta-AY.99.2..…...…14

| **Table S1.** Markov jumps inferred by discrete phylogeographic inference for Gamma-P1.Posterior mean estimates with 95% HPD intervals. Markov jumps associated with a Bayes factor support higher than three are in bold, corresponding a positive support transition realization, and zero values are represented with a hyphen. | | | |
| --- | --- | --- | --- |
| Start | Destination | Median | CI 95% HPD |
| Central-West | Central-West | - | - |
| Central-West | North | 6 | 3-9 |
| Central-West | Northeast | 4 | --9 |
| Central-West | South | 1 | 0-3 |
| Central-West | Southeast | 3 | --7 |
| Central-West | Study | 6 | 3-10 |
| North | Central-West | 11 | 6-16 |
| North | North | - | - |
| North | Northeast | **7** | **2-13** |
| North | South | - | - |
| North | Southeast | **5** | **0-10** |
| North | Study | **9** | **3-15** |
| Northeast | Central-West | 27 | 16-40 |
| Northeast | North | **24** | **13-37** |
| Northeast | Northeast | - | - |
| Northeast | South | **3** | **0-6** |
| Northeast | Southeast | **22** | **12-31** |
| Northeast | Study | **177** | **171-205** |
| South | Central-West | - | - |
| South | North | - | - |
| South | Northeast | - | - |
| South | South | - | - |
| South | Southeast | - | - |
| South | Study | - | 0-1 |
| Southeast | Central-West | 2 | 0-5 |
| Southeast | North | **2** | **1-5** |
| Southeast | Northeast | **7** | **3-10** |
| Southeast | South | - | - |
| Southeast | Southeast | - | - |
| Southeast | Study | **3** | **0-6** |
| Study | Central-West | 70 | 54-84 |
| Study | North | **66** | **49-82** |
| Study | Northeast | **94** | **68-113** |
| Study | South | **74** | **67-84** |
| Study | Southeast | **10** | **6-17** |
| Study | Study | - | - |

| **Table S2.** Summary of Markov jumps inferred for the Clusters of transmission for Gamma-P.1 in Parana in a discrete phylogeographic analysis at level of health administrative regions. Posterior mean estimates with 95% HPD intervals. Markov jumps associated with a Bayes factor support higher than three are in bold, corresponding a positive support transition realization, and zero values are represented with a hyphen. | | | |
| --- | --- | --- | --- |
| Start | Destination | Median | 95% HPD |
| Clade II |  |  |  |
| Cascavel | Cascavel | - | - |
| Cascavel | Foz do Iguacu | **2** | **0-6** |
| Cascavel | Curitiba | - | - |
| Cascavel | Guarapuava | - | 0-2 |
| Cascavel | Londrina | - | - |
| Cascavel | Maringa | - | - |
| Cascavel | Ponta Grossa | - | - |
| Foz do Iguacu | Cascavel | - | - |
| Foz do Iguacu | Foz do Iguacu | - | - |
| Foz do Iguacu | Curitiba | - | - |
| Foz do Iguacu | Guarapuava | - | - |
| Foz do Iguacu | Londrina | - | - |
| Foz do Iguacu | Maringa | - | - |
| Foz do Iguacu | Ponta Grossa | - | 0-1 |
| Curitiba | Cascavel | **16** | **8-19** |
| Curitiba | Foz do Iguacu | **19** | **11-24** |
| Curitiba | Curitiba | - | - |
| Curitiba | Guarapuava | **10** | **5-12** |
| Curitiba | Londrina | **5** | **0-6** |
| Curitiba | Maringa | **12** | **5-15** |
| Curitiba | Ponta Grossa | - | 0-1 |
| Guarapuava | Cascavel | - | 0-3 |
| Guarapuava | Foz do Iguacu | - | 0-1 |
| Guarapuava | Curitiba | - | - |
| Guarapuava | Guarapuava | - | - |
| Guarapuava | Londrina | - | - |
| Guarapuava | Maringa | - | 0-4 |
| Guarapuava | Ponta Grossa | - | 0-1 |
| Londrina | Cascavel | - | - |
| Londrina | Foz do Iguacu | - | - |
| Londrina | Curitiba | - | - |
| Londrina | Guarapuava | - | - |
| Londrina | Londrina | - | - |
| Londrina | Maringa | - | - |
| Londrina | Ponta Grossa | - | 0-1 |
| Maringa | Cascavel | - | 0-1 |
| Maringa | Foz do Iguacu | - | 0-2 |
| Maringa | Curitiba | - | - |
| Maringa | Guarapuava | - | 0-3 |
| Maringa | Londrina | - | - |
| Maringa | Maringa | - | - |
| Maringa | Ponta Grossa | - | 0-1 |
| Ponta Grossa | Cascavel | - | - |
| Ponta Grossa | Foz do Iguacu | - | - |
| Ponta Grossa | Curitiba | - | - |
| Ponta Grossa | Guarapuava | - | - |
| Ponta Grossa | Londrina | - | - |
| Ponta Grossa | Maringa | - | - |
| Ponta Grossa | Ponta Grossa | - | - |
|  |  |  |  |
| Clade III |  |  |  |
| Cascavel | Cascavel | - | - |
| Cascavel | Cornelio Procopio | - | - |
| Cascavel | Curitiba | - | - |
| Cascavel | Foz do Iguacu | - | 0-1 |
| Cascavel | Francisco Beltrao | - | 0-1 |
| Cascavel | Guarapuava | - | 0-1 |
| Cascavel | Londrina | - | - |
| Cascavel | Maringa | - | 0-3 |
| Cascavel | Ponta Grossa | - | - |
| Cornelio Procopio | Cascavel | - | - |
| Cornelio Procopio | Cornelio Procopio | - | - |
| Cornelio Procopio | Curitiba | - | - |
| Cornelio Procopio | Foz do Iguacu | - | - |
| Cornelio Procopio | Francisco Beltrao | - | - |
| Cornelio Procopio | Guarapuava | - | - |
| Cornelio Procopio | Londrina | - | - |
| Cornelio Procopio | Maringa | - | - |
| Cornelio Procopio | Ponta Grossa | - | - |
| Curitiba | Cascavel | **2** | **0-6** |
| Curitiba | Cornelio Procopio | - | - |
| Curitiba | Curitiba | - | - |
| Curitiba | Foz do Iguacu | - | 0-2 |
| Curitiba | Francisco Beltrao | - | 0-2 |
| Curitiba | Guarapuava | **0.33** | **0-2** |
| Curitiba | Londrina | - | 0-5 |
| Curitiba | Maringa | **2** | **0-5** |
| Curitiba | Ponta Grossa | - | - |
| Foz do Iguacu | Cascavel | - | - |
| Foz do Iguacu | Cornelio Procopio | - | - |
| Foz do Iguacu | Curitiba | - | - |
| Foz do Iguacu | Foz do Iguacu | - | - |
| Foz do Iguacu | Francisco Beltrao | - | - |
| Foz do Iguacu | Guarapuava | - | 0-1 |
| Foz do Iguacu | Londrina | - | - |
| Foz do Iguacu | Maringa | - | - |
| Foz do Iguacu | Ponta Grossa | - | - |
| Francisco Beltrao | Cascavel | - | 0-1 |
| Francisco Beltrao | Cornelio Procopio | - | - |
| Francisco Beltrao | Curitiba | - | - |
| Francisco Beltrao | Foz do Iguacu | - | 0-1 |
| Francisco Beltrao | Francisco Beltrao | - | - |
| Francisco Beltrao | Guarapuava | - | 0-1 |
| Francisco Beltrao | Londrina | - | - |
| Francisco Beltrao | Maringa | - | - |
| Francisco Beltrao | Ponta Grossa | - | - |
| Guarapuava | Cascavel | - | - |
| Guarapuava | Cornelio Procopio | - | - |
| Guarapuava | Curitiba | - | - |
| Guarapuava | Foz do Iguacu | - | 0-1 |
| Guarapuava | Francisco Beltrao | - | - |
| Guarapuava | Guarapuava | - | - |
| Guarapuava | Londrina | - | - |
| Guarapuava | Maringa | - | - |
| Guarapuava | Ponta Grossa | - | - |
| Londrina | Cascavel | - | 0-4 |
| Londrina | Cornelio Procopio | - | - |
| Londrina | Curitiba | **10** | **0-14** |
| Londrina | Foz do Iguacu | - | 0-1 |
| Londrina | Francisco Beltrao | - | 0-1 |
| Londrina | Guarapuava | - | 0-1 |
| Londrina | Londrina | - | - |
| Londrina | Maringa | **1** | **0-5** |
| Londrina | Ponta Grossa | - | - |
| Maringa | Cascavel | **1** | **0-4** |
| Maringa | Cornelio Procopio | - | - |
| Maringa | Curitiba | - | - |
| Maringa | Foz do Iguacu | - | 0-1 |
| Maringa | Francisco Beltrao | - | 0-1 |
| Maringa | Guarapuava | - | 0-1 |
| Maringa | Londrina | - | - |
| Maringa | Maringa | - | - |
| Maringa | Ponta Grossa | - | - |
| Ponta Grossa | Cascavel | - | - |
| Ponta Grossa | Cornelio Procopio | - | - |
| Ponta Grossa | Curitiba | - | - |
| Ponta Grossa | Foz do Iguacu | - | - |
| Ponta Grossa | Francisco Beltrao | - | - |
| Ponta Grossa | Guarapuava | - | - |
| Ponta Grossa | Londrina | - | - |
| Ponta Grossa | Maringa | - | - |
| Ponta Grossa | Ponta Grossa | - | - |
|  |  |  |  |
| Clade IV |  |  |  |
| Curitiba | Curitiba | - | - |
| Curitiba | Guarapuava | **1** | **0-1** |
| Curitiba | Londrina | **2** | **0-3** |
| Curitiba | Maringa | **3** | **0-5** |
| Guarapuava | Curitiba | - | - |
| Guarapuava | Guarapuava | - | - |
| Guarapuava | Londrina | - | - |
| Guarapuava | Maringa | - | - |
| Londrina | Curitiba | - | 0-1 |
| Londrina | Guarapuava | - | 0-1 |
| Londrina | Londrina | - | - |
| Londrina | Maringa | - | 0-3 |
| Maringa | Curitiba | - | 0-2 |
| Maringa | Guarapuava | - | 0-1 |
| Maringa | Londrina | - | 0-2 |
| Maringa | Maringa | - | - |
|  |  |  |  |
| Clade V |  |  |  |
| Cascavel | Cascavel | - | - |
| Cascavel | Cornelio Procopio | - | - |
| Cascavel | Curitiba | - | 0-1 |
| Cascavel | Foz do Iguacu | - | - |
| Cascavel | Francisco Beltrao | - | - |
| Cascavel | Guarapuava | - | 0-1 |
| Cascavel | Londrina | - | - |
| Cascavel | Maringa | - | 0-1 |
| Cascavel | Ponta Grossa | - | - |
| Cornelio Procopio | Cascavel | - | - |
| Cornelio Procopio | Cornelio Procopio | - | - |
| Cornelio Procopio | Curitiba | - | - |
| Cornelio Procopio | Foz do Iguacu | - | - |
| Cornelio Procopio | Francisco Beltrao | - | - |
| Cornelio Procopio | Guarapuava | - | - |
| Cornelio Procopio | Londrina | - | - |
| Cornelio Procopio | Maringa | - | - |
| Cornelio Procopio | Ponta Grossa | - | - |
| Curitiba | Cascavel | **7** | **5-9** |
| Curitiba | Cornelio Procopio | - | 0-1 |
| Curitiba | Curitiba | - | - |
| Curitiba | Foz do Iguacu | 3 | 0-5 |
| Curitiba | Francisco Beltrao | - | 0-1 |
| Curitiba | Guarapuava | **8** | **6-10** |
| Curitiba | Londrina | **5** | **3-10** |
| Curitiba | Maringa | **9** | **4-13** |
| Curitiba | Ponta Grossa | 5 | 3-6 |
| Foz do Iguacu | Cascavel | - | 0-1 |
| Foz do Iguacu | Cornelio Procopio | - | - |
| Foz do Iguacu | Curitiba | 2 | 1-4 |
| Foz do Iguacu | Foz do Iguacu | - | - |
| Foz do Iguacu | Francisco Beltrao | 2 | 0-3 |
| Foz do Iguacu | Guarapuava | - | 0-1 |
| Foz do Iguacu | Londrina | - | 0-1 |
| Foz do Iguacu | Maringa | 1 | 0-2 |
| Foz do Iguacu | Ponta Grossa | - | - |
| Francisco Beltrao | Cascavel | - | - |
| Francisco Beltrao | Cornelio Procopio | - | - |
| Francisco Beltrao | Curitiba | - | - |
| Francisco Beltrao | Foz do Iguacu | - | 0-2 |
| Francisco Beltrao | Francisco Beltrao | - | - |
| Francisco Beltrao | Guarapuava | - | - |
| Francisco Beltrao | Londrina | - | - |
| Francisco Beltrao | Maringa | - | - |
| Francisco Beltrao | Ponta Grossa | - | - |
| Guarapuava | Cascavel | - | 0-1 |
| Guarapuava | Cornelio Procopio | - | - |
| Guarapuava | Curitiba | - | 0-2 |
| Guarapuava | Foz do Iguacu | - | - |
| Guarapuava | Francisco Beltrao | - | - |
| Guarapuava | Guarapuava | - | - |
| Guarapuava | Londrina | - | - |
| Guarapuava | Maringa | - | 0-1 |
| Guarapuava | Ponta Grossa | - | 0-1 |
| Londrina | Cascavel | - | 0-3 |
| Londrina | Cornelio Procopio | 2 | 1-2 |
| Londrina | Curitiba | **24** | **15-35** |
| Londrina | Foz do Iguacu | 1 | 0-2 |
| Londrina | Francisco Beltrao | 1 | 0-1 |
| Londrina | Guarapuava | - | 0-1 |
| Londrina | Londrina | - | - |
| Londrina | Maringa | **12** | **8-16** |
| Londrina | Ponta Grossa | - | 0-1 |
| Maringa | Cascavel | - | 0-1 |
| Maringa | Cornelio Procopio | - | - |
| Maringa | Curitiba | - | 0-2 |
| Maringa | Foz do Iguacu | - | 0-1 |
| Maringa | Francisco Beltrao | - | - |
| Maringa | Guarapuava | - | - |
| Maringa | Londrina | - | 0-2 |
| Maringa | Maringa | - | - |
| Maringa | Ponta Grossa | - | 0-1 |
| Ponta Grossa | Cascavel | - | - |
| Ponta Grossa | Cornelio Procopio | - | - |
| Ponta Grossa | Curitiba | - | 0-1 |
| Ponta Grossa | Foz do Iguacu | - | - |
| Ponta Grossa | Francisco Beltrao | - | - |
| Ponta Grossa | Guarapuava | - | 0-1 |
| Ponta Grossa | Londrina | - | - |
| Ponta Grossa | Maringa | - | 0-1 |
| Ponta Grossa | Ponta Grossa | - | - |

| **Table S3.** Markov jumps inferred by discrete phylogeographic inference for Delta-AY.101.Posterior mean estimates with 95% HPD intervals. Markov jumps associated with a Bayes factor support higher than three are in bold, corresponding a positive support transition realization, and zero values are represented with a hyphen. | | | | |  |
| --- | --- | --- | --- | --- | --- |
| Start | Destination | Median | CI 95% HPD | | |
| Central America | Central America | - | - |  | |
| Central America | Central-West | - | - |  | |
| Central America | Europe | - | - |  | |
| Central America | North | - | - |  | |
| Central America | North America | - | - |  | |
| Central America | Northeast | - | - |  | |
| Central America | Parana | - | - |  | |
| Central America | South | 1 | 1-1 |  | |
| Central America | South America | 1 | 1-1 |  | |
| Central America | Southeast | - | - |  | |
| Central America | Study | - | - |  | |
| Central-West | Central America | - | - |  | |
| Central-West | Central-West | - | - |  | |
| Central-West | Europe | - | - |  | |
| Central-West | North | 1 | 0-1 |  | |
| Central-West | North America | - | - |  | |
| Central-West | Northeast | 4 | 4-5 |  | |
| Central-West | Parana | 9 | 7-12 |  | |
| Central-West | South | 4 | 3-5 |  | |
| Central-West | South America | 4 | 2-5 |  | |
| Central-West | Southeast | 4 | 3-7 |  | |
| Central-West | Study | - | 0-1 |  | |
| Europe | Central America | - | - |  | |
| Europe | Central-West | - | - |  | |
| Europe | Europe | - | - |  | |
| Europe | North | - | - |  | |
| Europe | North America | - | - |  | |
| Europe | Northeast | - | - |  | |
| Europe | Parana | - | - |  | |
| Europe | South | **1** | **0-1** |  | |
| Europe | South America | - | - |  | |
| Europe | Southeast | - | - |  | |
| Europe | Study | - | - |  | |
| North | Central America | - | - |  | |
| North | Central-West | - | - |  | |
| North | Europe | - | - |  | |
| North | North | - | - |  | |
| North | North America | - | - |  | |
| North | Northeast | - | - |  | |
| North | Parana | - | - |  | |
| North | South | - | - |  | |
| North | South America | - | - |  | |
| North | Southeast | **1** | **0-1** |  | |
| North | Study | - | - |  | |
| North America | Central America | 1 | 1-1 |  | |
| North America | Central-West | - | - |  | |
| North America | Europe | 1 | 1-1 |  | |
| North America | North | - | - |  | |
| North America | North America | - | - |  | |
| North America | Northeast | - | - |  | |
| North America | Parana | - | - |  | |
| North America | South | - | - |  | |
| North America | South America | - | - |  | |
| North America | Southeast | - | - |  | |
| North America | Study | - | - |  | |
| Northeast | Central America | - | - |  | |
| Northeast | Central-West | - | - |  | |
| Northeast | Europe | - | - |  | |
| Northeast | North | - | 0-1 |  | |
| Northeast | North America | 1 | 1-1 |  | |
| Northeast | Northeast | - | - |  | |
| Northeast | Parana | - | - |  | |
| Northeast | South | **3** | **3-3** |  | |
| Northeast | South America | 1 | 1-2 |  | |
| Northeast | Southeast | **2** | **2-4** |  | |
| Northeast | Study | - | - |  | |
| Parana | Central America | - | - |  | |
| Parana | Central-West | 2 | 2-4 |  | |
| Parana | Europe | - | - |  | |
| Parana | North | **1** | **1-1** |  | |
| Parana | North America | - | - |  | |
| Parana | Northeast | - | - |  | |
| Parana | Parana | - | - |  | |
| Parana | South | - | - |  | |
| Parana | South America | - | - |  | |
| Parana | Southeast | **2** | **2-4** |  | |
| Parana | Study | - | - |  | |
| South | Central America | - | - |  | |
| South | Central-West | 6 | 5-7 |  | |
| South | Europe | **2** | **2-5** |  | |
| South | North | **14** | **14-15** |  | |
| South | North America | - | - |  | |
| South | Northeast | **7** | **6-7** |  | |
| South | Parana | **16** | **13-18** |  | |
| South | South | - | - |  | |
| South | South America | 4 | 2-5 |  | |
| South | Southeast | **20** | **17-22** |  | |
| South | Study | **4** | **2-4** |  | |
| South America | Central America | 3 | 3-3 |  | |
| South America | Central-West | - | 0-1 |  | |
| South America | Europe | 5 | 5-5 |  | |
| South America | North | - | - |  | |
| South America | North America | 3 | 3-3 |  | |
| South America | Northeast | - | - |  | |
| South America | Parana | 5 | 4-6 |  | |
| South America | South | - | - |  | |
| South America | South America | - | - |  | |
| South America | Southeast | 2 | 2-2 |  | |
| South America | Study | - | - |  | |
| Southeast | Central America | - | - |  | |
| Southeast | Central-West | 5 | --7 |  | |
| Southeast | Europe | - | - |  | |
| Southeast | North | - | - |  | |
| Southeast | North America | 2 | 2-2 |  | |
| Southeast | Northeast | **2** | **2-4** |  | |
| Southeast | Parana | **6** | **3-9** |  | |
| Southeast | South | **2** | **1-4** |  | |
| Southeast | South America | 2 | 1-3 |  | |
| Southeast | Southeast | - | - |  | |
| Southeast | Study | - | - |  | |
| Study | Central America | - | - |  | |
| Study | Central-West | 3 | 1-5 |  | |
| Study | Europe | **4** | **2-5** |  | |
| Study | North | - | - |  | |
| Study | North America | - | - |  | |
| Study | Northeast | **8** | **6-8** |  | |
| Study | Parana | **8** | **7-11** |  | |
| Study | South | **7** | **5-8** |  | |
| Study | South America | 3 | 3-4 |  | |
| Study | Southeast | **19** | **17-21** |  | |
| Study | Study | - | - |  | |
| Central America | Central America | - | - |  | |

| **Table S4.** Summary of Markov jumps inferred for the Clusters of transmission for Delta-AY.101 in Parana in a discrete phylogeographic analysis at level of health administrative regions. Posterior mean estimates with 95% HPD intervals. Markov jumps associated with a Bayes factor support higher than three are in bold, corresponding a positive support transition realization, and zero values are represented with a hyphen. | | | |
| --- | --- | --- | --- |
| Start | Destination | Median | 95% HPD |
| Clade I |  |  |  |
| Curitiba | Curitiba | - | - |
| Curitiba | Londrina | **1** | **0-2** |
| Curitiba | Maringa | **2** | **0-3** |
| Londrina | Curitiba | - | - |
| Londrina | Londrina | - | - |
| Londrina | Maringa | - | 0-1 |
| Maringa | Curitiba | - | 0-2 |
| Maringa | Londrina | - | 0-1 |
| Maringa | Maringa | - | - |
|  |  |  |  |
| Clade II |  |  |  |
| Colombo | Colombo | - | - |
| Colombo | Curitiba | - | - |
| Colombo | Foz do Iguacu | - | 0-1 |
| Colombo | Guarapuava | - | - |
| Colombo | Londrina | - | - |
| Colombo | Maringa | - | - |
| Curitiba | Colombo | - | 0-1 |
| Curitiba | Curitiba | - | - |
| Curitiba | Foz do Iguacu | - | 0-1 |
| Curitiba | Guarapuava | **1** | **0-3** |
| Curitiba | Londrina | - | - |
| Curitiba | Maringa | - | - |
| Foz do Iguacu | Colombo | - | 0-1 |
| Foz do Iguacu | Curitiba | - | - |
| Foz do Iguacu | Foz do Iguacu | - | - |
| Foz do Iguacu | Guarapuava | - | - |
| Foz do Iguacu | Londrina | - | - |
| Foz do Iguacu | Maringa | - | - |
| Guarapuava | Colombo | - | 0-1 |
| Guarapuava | Curitiba | - | - |
| Guarapuava | Foz do Iguacu | - | 0-1 |
| Guarapuava | Guarapuava | - | - |
| Guarapuava | Londrina | - | - |
| Guarapuava | Maringa | - | - |
| Londrina | Colombo | - | 0-1 |
| Londrina | Curitiba | **11** | **8-13** |
| Londrina | Foz do Iguacu | - | 0-1 |
| Londrina | Guarapuava | **1** | **0-3** |
| Londrina | Londrina | - | - |
| Londrina | Maringa | **7** | **5-8** |
| Maringa | Colombo | - | 0-1 |
| Maringa | Curitiba | - | - |
| Maringa | Foz do Iguacu | - | 0-1 |
| Maringa | Guarapuava | - | 0-1 |
| Maringa | Londrina | - | - |
| Maringa | Maringa | - | - |

| **Table S5.** Markov jumps inferred by discrete phylogeographic inference for Delta-AY.99.2.Posterior mean estimates with 95% HPD intervals. Markov jumps associated with a Bayes factor support higher than three are in bold, corresponding a positive support transition realization, and zero values are represented with a hyphen. | | | |
| --- | --- | --- | --- |
| Start | Destination | Median | 95% HPD |
| Asia | Asia | - | - |
| Asia | Central-West | - | - |
| Asia | Europe | - | - |
| Asia | North | - | - |
| Asia | North America | - | - |
| Asia | Northeast | - | - |
| Asia | South | - | - |
| Asia | South America | - | - |
| Asia | Southeast | - | - |
| Asia | Study | - | - |
| Central-West | Asia | 1 | 1-1 |
| Central-West | Central-West | - | - |
| Central-West | Europe | - | - |
| Central-West | North | 6 | 4-9 |
| Central-West | North America | - | - |
| Central-West | Northeast | 8 | 5-9 |
| Central-West | South | 5 | 4-6 |
| Central-West | South America | - | - |
| Central-West | Southeast | 5 | 4-6 |
| Central-West | Study | 1 | 1-2 |
| Europe | Asia | - | - |
| Europe | Central-West | - | - |
| Europe | Europe | - | - |
| Europe | North | - | - |
| Europe | North America | - | - |
| Europe | Northeast | - | - |
| Europe | South | - | - |
| Europe | South America | - | - |
| Europe | Southeast | - | - |
| Europe | Study | - | - |
| North | Asia | **1** | **1-1** |
| North | Central-West | 7 | 3-8 |
| North | Europe | **2** | **2-2** |
| North | North | - | - |
| North | North America | 2 | 1-3 |
| North | Northeast | 5 | 5-6 |
| North | South | **2** | **2-3** |
| North | South America | 2 | 2-3 |
| North | Southeast | - | - |
| North | Study | - | - |
| North America | Asia | - | - |
| North America | Central-West | - | - |
| North America | Europe | - | - |
| North America | North | 1 | 1-2 |
| North America | North America | - | - |
| North America | Northeast | 1 | 0-1 |
| North America | South | - | - |
| North America | South America | - | - |
| North America | Southeast | - | - |
| North America | Study | - | - |
| Northeast | Asia | - | - |
| Northeast | Central-West | 8 | 5-11 |
| Northeast | Europe | **2** | **2-3** |
| Northeast | North | **1** | **0-2** |
| Northeast | North America | 1 | 0-3 |
| Northeast | Northeast | - | - |
| Northeast | South | **8** | **4-11** |
| Northeast | South America | 4 | 2-4 |
| Northeast | Southeast | **10** | **5-16** |
| Northeast | Study | - | - |
| South | Asia | - | - |
| South | Central-West | 5 | 3-6 |
| South | Europe | - | - |
| South | North | **4** | **3-5** |
| South | North America | 2 | 1-3 |
| South | Northeast | **11** | **9-13** |
| South | South | - | - |
| South | South America | 5 | 3-5 |
| South | Southeast | **1** | **1-2** |
| South | Study | **9** | **5-13** |
| South America | Asia | - | - |
| South America | Central-West | - | - |
| South America | Europe | - | - |
| South America | North | - | 0-1 |
| South America | North America | - | - |
| South America | Northeast | - | - |
| South America | South | - | - |
| South America | South America | - | - |
| South America | Southeast | - | - |
| South America | Study | - | - |
| Southeast | Asia | - | - |
| Southeast | Central-West | 26 | 22-29 |
| Southeast | Europe | **3** | **2-3** |
| Southeast | North | **27** | **25-29** |
| Southeast | North America | 4 | 4-5 |
| Southeast | Northeast | **65** | **54-72** |
| Southeast | South | **32** | **28-36** |
| Southeast | South America | - | 0-4 |
| Southeast | Southeast | - | - |
| Southeast | Study | **12** | **10-15** |
| Study | Asia | - | - |
| Study | Central-West | - | - |
| Study | Europe | - | - |
| Study | North | - | - |
| Study | North America | - | - |
| Study | Northeast | - | - |
| Study | South | **8** | **5-12** |
| Study | South America | - | - |
| Study | Southeast | - | - |
| Study | Study | - | - |
|  |  |  |  |


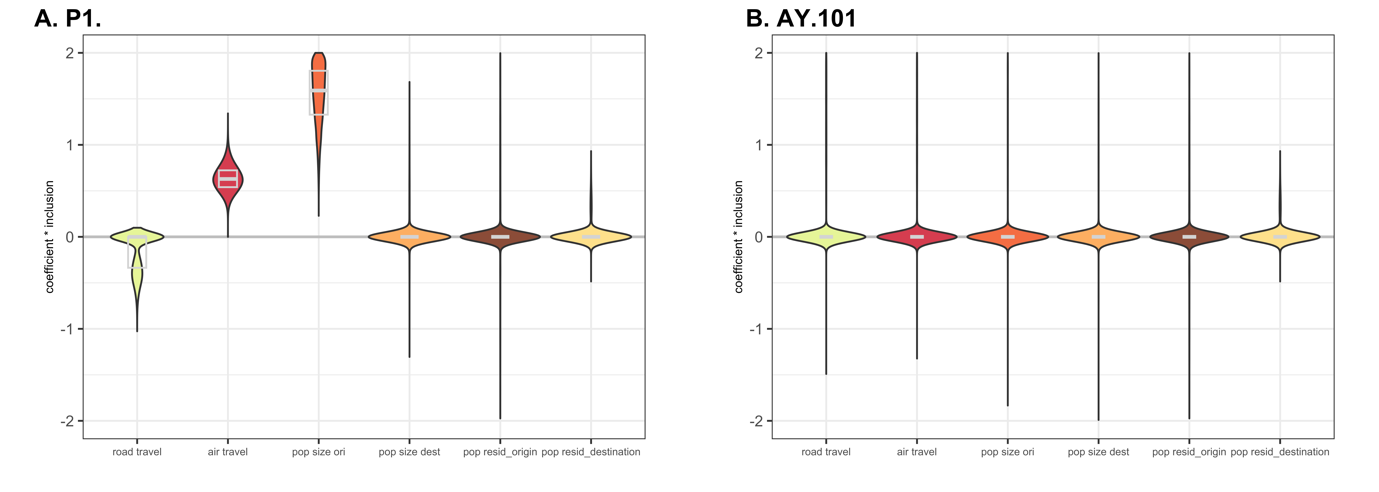


**Figure 1.** **Predictors of SARS-CoV-2 lineage movements using a phylogeographic GLM-diffusion model.** The plots summarize the posterior distribution of the product of the coefficient (on a log scale) and the inclusion probability for the predictors (coefficient * Inclusion), for the Gamma and Delta lineages (median and quantile estimates), respectively. The predictors tested were road travel, air travel, population size at the origin and destination, and the residuals for a regression of population size and genomes numbers at the origin and destination. The phylogeographic analysis used cities' health administrative regions as discrete geographic units.
